# A Water‐Soluble Tetraazaperopyrene Dye as Strong G‐Quadruplex DNA Binder

**DOI:** 10.1002/chem.201504934

**Published:** 2016-03-21

**Authors:** Lena Hahn, Niklaas J. Buurma, Lutz H. Gade

**Affiliations:** ^1^ Anorganisch-Chemisches-InstitutUniversität HeidelbergIm Neuenheimer Feld 27069120HeidelbergGermany), Fax; ^2^ Physical Organic Chemistry CentreSchool of ChemistryCardiff University, Main BuildingPark PlaceCardiffCF10 3ATUK), Fax

**Keywords:** DNA, docking studies, dyes/pigments, G-quadruplexes

## Abstract

The interactions of the water‐soluble tetraazaperopyrene dye **1** with ct‐DNA, duplex‐[(dAdT)_12_ **⋅**(dAdT)_12_], duplex‐[(dGdC)_12_ **⋅**(dGdC)_12_] as well as with two G‐quadruplex‐forming sequences, namely the human telomeric 22AG and the promotor sequence c‐myc, were investigated by means of UV/visible and fluorescence spectroscopy, isothermal titration calorimetry (ITC) and molecular docking studies. Dye **1** exhibits a high affinity for G‐quadruplex structures over duplex DNA structures. Furthermore, the ligand shows promising G‐quadruplex discrimination, with an affinity towards c‐myc of 2×10^7^ m ^−1^ (i.e., *K*_d_=50 nm), which is higher than for 22AG (4×10^6^ m ^−1^). The ITC data reveal that compound **1** interacts with c‐myc in a stoichiometric ratio of 1:1 but also indicate the presence of two identical lower affinity secondary binding sites per quadruplex. In 22AG, there are two high affinity binding sites per quadruplex, that is, one on each side, with a further four weaker binding sites. For both quadruplex structures, the high affinity interactions between compound **1** and the quadruplex‐forming nucleic acid structures are weakly endothermic. Molecular docking studies suggest an end‐stacking binding mode for compound **1** interacting with quadruplex structures, and a higher affinity for the parallel conformation of c‐myc than for the mixed‐hybrid conformation of 22AG. In addition, docking studies also suggest that the reduced affinity for duplex DNA structures is due to the non‐viability of an intercalative binding mode.

## Introduction

Single strands of DNA containing repeats of guanine bases can form Hoogsteen hydrogen bonds between four guanosine residues, resulting in so‐called G‐tetrads.^1^ Stacking of several G‐tetrads leads to the typical G‐quadruplex structure, which is stabilised by chelation of a metal cation (usually K^+^ or Na^+^).^2^, ^3^

Single‐stranded guanine‐rich DNA sequences are commonly found within telomeres, which are the non‐coding ends of chromatids that protect the genes adjacent to them.^4^, ^5^, ^6^, ^7^, ^8^, ^9^ In addition, guanine‐rich sequences were also found in various promotor regions.^10^ There are as many as 71 600 sequences that have been identified as potentially quadruplex‐forming sequences in the genome^11^ and quadruplex formation has been suggested to be of relevance in genome integrity, transcription, epigenetic regulation and meiosis.^12^, ^13^

Historically, G‐quadruplex structures have attracted significant interest as a target in attempts to control cancer proliferation. These attempts are based on the observation that apoptosis normally occurs due to a critical shortening of the telomeres as a result of repeated cellular replication cycles. In their unfolded form, telomeres serve as primers for the enzyme telomerase, which is responsible for maintaining the telomeric length throughout cellular replication. Telomerase remains mostly inactive in normal somatic cells but is expressed by 85–90 % of cancer cells. Overexpression of telomerase thus effectively prohibits apoptosis, and therefore renders cancer cells immortal. Controlling telomerase interaction with the telomeric ends of chromosomes by driving quadruplex folding through added quadruplex binders has therefore become a popular approach in attempts to control cancer proliferation. Indeed, G‐quadruplex formation has been shown to hinder telomerase activity, but in addition it is implicated in the regulation of gene expression and quadruplex structures have thus become a potential target in oncology.^14^, ^15^ A range of small molecules has been shown to strongly bind and stabilise such G‐quadruplex structures, thereby rendering these compounds potential anticancer drugs.^10^, ^16^, ^17^, ^18^, ^19^, ^20^, ^21^, ^22^, ^23^, ^24^, ^25^, ^26^ Recent findings, such as the fact that parallel quadruplexes can still act as a substrate for telomerase,^27^ suggest, however, that indirect telomerase inhibition is not necessarily the dominant contribution in anticancer activity of quadruplex‐binding ligands.

In addition to a potential use in therapy, quadruplex binders are of interest in the development of biosensors and imaging techniques. In imaging, turn‐on fluorescent probes^28^, ^29^, ^30^, ^31^, ^32^, ^33^ have become popular although fluorescence quenchers remain useful in, for example, Förster resonance energy transfer (FRET) pairs.^34^ For biosensors, both spectroscopic and electrochemical properties are of interest. Finally, nucleic acid structures also form interesting building blocks for directed assembly of nanostructures.^35^

To have potential as therapeutic agents, as biosensors and in bioimaging or in directed assembly, quadruplex binders have to meet certain criteria. One criterion is a strong selectivity for G‐quadruplexes over double‐stranded DNA, which is often found to be a problem when developing quadruplex‐binding agents. Currently known G‐quadruplex binders are often polycyclic aromatic compounds.^22^, ^36^, ^37^ The optoelectronic properties of such compounds render them suitable for biosensing and imaging in addition to their potential therapeutic use.^33^, ^35^, ^38^, ^39^, ^40^ Recently, we reported a new class of polyheterocyclic aromatics, that is, 1,3,8,10‐tetraazaperopyrenes (TAPPs).^41^, ^42^, ^43^, ^44^ Besides giving rise to promising results in the field of organic electronics,^45^, ^46^, ^47^, ^48^ it was shown that water‐ soluble derivatives can be employed as fluorescence probes selectively staining cell nuclei.^50^ The structure of TAPPs is reminiscent of perylene diimides, which are known to be good quadruplex binders,^49^, ^50^ such as, for example, PIPER.^51^ TAPP derivatives therefore appear promising for biosensing and imaging applications, as well as for a biopolymer‐directed assembly of functional nanostructures.

**Figure 1:**
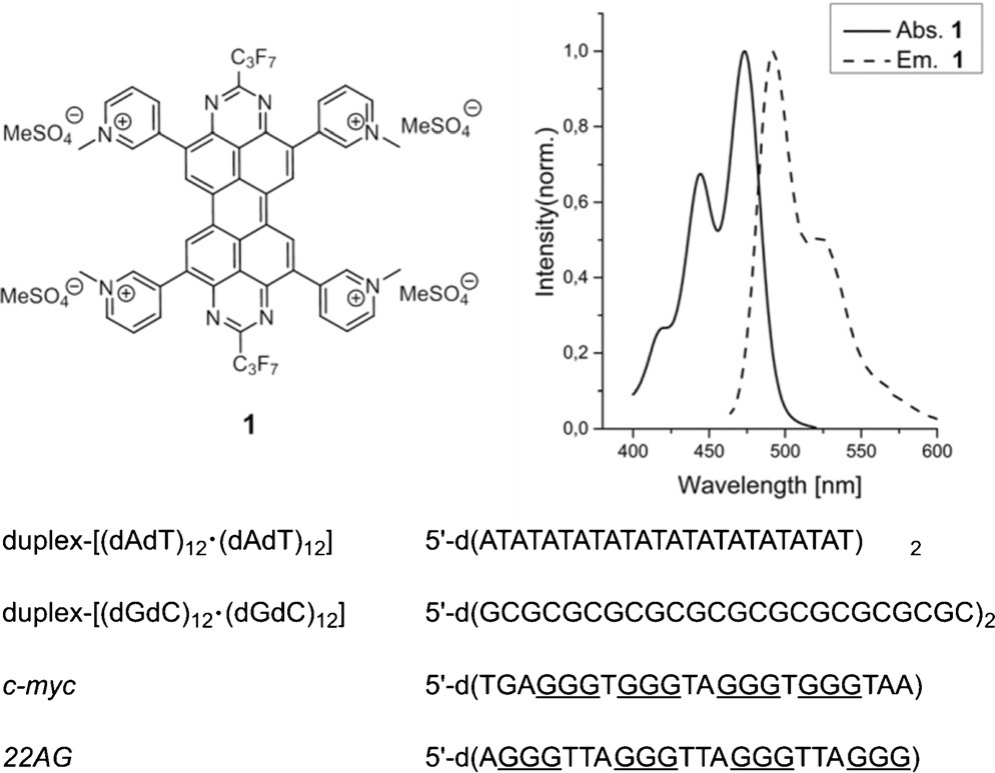
Top) Absorption and emission band for compound **1** recorded in water.^47^ Bottom) Synthetic oligonucleotide sequences used in this work.

To develop these applications, the interactions of the TAPP derivative **1** (Figure 1) with a relatively small group of nucleic acid structures were studied in detail. Herein, we report our studies of the interactions of the water‐soluble TAPP **1** with calf thymus DNA, duplex‐[(dAdT)_12_ **⋅**(dAdT)_12_] and duplex‐[(dGdC)_12_ **⋅**(dGdC)_12_], as well as the interactions with two different G‐quadruplex‐forming DNA sequences, namely the human telomeric sequence 22AG and the promotor sequence c‐myc (Figure 1, bottom), by means of UV/visible and fluorescence spectroscopy as well as isothermal titration calorimetry (ITC) and molecular docking studies.

## Results and Discussion

The synthesis of compound **1** has previously been reported, its absorption and emission spectra in water are shown in Figure 1 top.^47^ The heptafluoropropyl substituents were introduced to the tetraazaperopyrene structure to stabilise the fluorophore and in order to enhance its solubility in organic solvents. This construction principle was established in our group over the past years.^41^, ^42^, ^43^, ^44^, ^45^, ^46^, ^47^, ^48^ Water solubility of compound **1** was achieved by functionalisation of the TAPP core with pyridyl substituents followed by methylation of the pyridyl nitrogen atoms and optimised by variation of the counter anions.^47^ Compound **1** displays a characteristic π*←π absorption band with a maximum at *λ*=474 nm and a corresponding emission band (max. *λ*=493 nm; fluorescence quantum yield in water is 82 %).^47^

### Comments on the structures of c‐myc and 22AG under the experimental conditions of this study

We note that c‐myc forms a relatively stable parallel structure, whereas the human telomeric sequence studied in this work gives rise to mixed hybrid structures under the experimental conditions.^52^, ^53^ Moreover, for both c‐myc^54^ and 22AG^55^ the unfolding kinetics are slow compared to the experimental timescales of the techniques used here, implying that binding is unlikely to drive changes from one type of structure to another on the timescale of the experiments.^56^

The circular dichroism (CD) spectrum for 22AG under the experimental conditions was recorded (Figure S5 a in the Supporting Information) and is in agreement with the reported spectrum for the mixed‐hybrid quadruplex structure.^53^, ^57^ The significant spectral features do not change in the presence of compound **1** (Figure S5 b in the Supporting Information), indicating that 22AG retains its mixed‐hybrid structure in the presence of compound **1**, at least under the conditions and on the timescale of our experiments.

### Interaction of compound 1 with duplex and quadruplex DNA

#### UV/visible and fluorescence titrations

**Figure 2:**
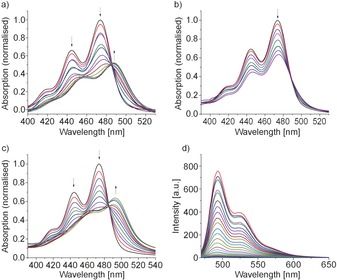
Absorption spectra of compound **1** in MOPS buffer in the presence of a) ct‐DNA, b) duplex‐[(dAdT)_12_ **⋅**(dAdT)_12_] and c) c‐myc. d) Fluorescence spectra for compound **1** in the presence of c‐myc. For the other spectra see the Supporting Information (Figures S1 a–c).

Interactions of compound **1** with the nucleic acid structures were first studied by means of UV/visible and fluorescence spectroscopy. The nucleic acid solutions were added stepwise to a 2 μm solution of compound **1** in a buffer (25 mm 3‐(*N*‐morpholino)‐propanesulfonic acid (MOPS), 100 mm KCl, 1 mm ethylenediaminetetraacetic acid (EDTA), pH 7.1) and the absorption spectra were recorded after each addition (Figure 2). The titrations with all five nucleic acid structures result in a hypochromic effect on the π*←π absorption band at *λ*=474 nm along with a bathochromic shift of the absorption maximum. For the titrations with ct‐DNA and 22AG the absorption maximum of TAPP **1** is red shifted by 12 nm, the absorption shift of 9 nm for the titration with duplex‐[(dGdC)_12_ **⋅**(dGdC)_12_] is smaller, whereas the titration with the c‐myc quadruplex results in the greatest red shift of 18 nm. On the other hand, the titration with duplex‐[(dAdT)_12_ **⋅**(dAdT)_12_] barely shifted the absorption maximum (4 nm). The hypochromism was approximately 50 % for the interaction of TAPP with ct‐DNA, duplex‐[(dGdC)_12_ **⋅**(dGdC)_12_], 22AG and c‐myc, but lower for interaction with duplex‐[(dAdT)_12_ **⋅**(dAdT)_12_].

Similarly, fluorescence titrations were carried out by stepwise addition of the nucleic acid structures to a 2 μm solution of compound **1**, under conditions identical to those used for the UV/visible titrations. Titrations with all five nucleic acid structures led to quenching of the fluorescence with no shift of the emission maximum. Figure 2 d shows the emission spectra for the titration of compound **1** with c‐myc. The decrease of the emission intensity depends on the added nucleic acid structure. In all cases, the apparent fluorescence quenching, *H*_em, app_, upon full binding of compound **1** exceeds the hypochromicity *H*_abs_, indicating that the decrease in the emission is not only the result of the decrease in the absorbance of compound **1** at the excitation wavelength (*λ*=460 nm). Furthermore, the decrease in the emission intensity upon addition of two equivalents of the nucleic acid structures also strongly depends on the added nucleic acid structure (Figure S1 b in the Supporting Information), and only the titration of compound **1** with c‐myc resulted in complete quenching of the fluorescence. Table 1 gives an overview of the spectroscopic data.

**Table 1:** Spectroscopic data for the titration of compound **1** with the different nucleic acid structures.

	*λ*_max, bound_	Δ*λ* [nm]	*H*_abs_^[a]^ (*λ*=474 nm) [%]	*H*_em, app_^[b]^ (*λ*=493 nm) [%]
ct‐DNA	486	12	(47.2±1.3)	(85.1±2.6)
(dAdT)_12_ **⋅**(dAdT)_12_	478	4	(40.0±1.7)	(70.3±2.2)
(dGdC)_12_ **⋅**(dGdC)_12_	483	9	(49.5±2.1)	(95.1±1.6)
22AG	486	12	(48.1±1.9)	(83.8±3.1)
c‐myc	492	18	(50.6±2.0)	(99.0±0.8)

[a] *H*_abs_ is the change in the extinction coefficient upon binding defined as (−Δ_binding_ *ɛ*_474 nm_/*ɛ*_474 nm, free_)×100 % where −Δ_binding_ *ɛ*_474 nm_ corresponds to *ɛ*_474 nm, free_−*ɛ*_474 nm, bound_, also see Eq. (S.1) in the Supporting Information. [b] *H*_em, app_=(intensity free_493 nm_−intensity bound_493 nm_)/(intensity free_493 nm_).

The hypochromism and the red shift of the absorption maximum, as well as the quenching of the fluorescence, indicate interaction between TAPP **1** and the DNA sequences. The quenching of the fluorescence of compound **1** might be considered a disadvantage in comparison with turn‐on probes for bioimaging of nucleic acid structures. However, there is scope for the use of a quadruplex‐selective quencher in FRET pairs, for example in combination with the well‐known duplex‐DNA minor‐groove binder H33258 (or one of its derivatives) that has the required fluorescence characteristics, which would allow the detection of double‐stranded DNA adjacent to quadruplex DNA. In addition, the electrochemical properties of compound **1** make it an interesting sensitizer in electrochemical biosensors and the use as a building block in nucleic acid based nanostructures does not necessarily require fluorescent properties of the binder.

In order to gain further insight into the binding mode and to compare the affinity of compound **1** for each DNA sequence quantitatively, plots of the absorbance at *λ*=474 nm and the fluorescence emission as a function of the nucleic acid concentration were constructed (Figure S1 in the Supporting Information). The binding site size in terms of base pairs (for duplex structures) or in terms of tetrads (for quadruplex structures), *n*, as well as the apparent binding constant *K*_b_ were determined for each sequence at 25 °C by fitting a multiple independent binding sites (MIS) model, which corrects for ligand dilution (see Eq. (S.1) in the Supporting Information), to the data.^58^ Table 2 gives an overview of the affinity constants *K*_b_ and the binding site sizes *n* (expressed in numbers of base pairs/tetrads per binding site) for each sequence. The binding parameters show that compound **1** displays a high affinity for duplex‐[(dGdC)_12_ **⋅**(dGdC)_12_], whereas the affinity for duplex‐[(dAdT)_12_ **⋅**(dAdT)_12_] is negligible. Analysis of the data for compound **1** interacting with ct‐DNA in terms of the MIS model, despite the heterogeneity of the binding sites, gives an apparent affinity in the range of that obtained for duplex‐[(dGdC)_12_ **⋅**(dGdC)_12_]. Finally and notably, comparison of the *K*_b_ values obtained for duplex‐[(dGdC)_12_ **⋅**(dGdC)_12_] with the binding parameters obtained for the quadruplex sequences demonstrates that the affinity towards quadruplex structures is 4 to 10 times higher than towards duplex DNA.

**Table 2:** Binding constants and stoichiometries for TAPP 1 interacting with different DNA structures in 25 mm MOPS, 100 mm KCl, 1 mm EDTA, pH 7.1 at 20 °C.

	*K*_b_(UV/Vis) [10^6^×m ^−1^]	*n*(UV/Vis)^[a]^ [BP]/[TAPP] [T]/TAPP	*K*_b_(Fl.) [10^6^×m ^−1^]	*n*(FL.)^[a]^ [BP]/[TAPP] [T]/TAPP
ct‐DNA	(2.1±0.5)	(6.3±0.5)	(1.6±0.2)	(5.9±0.2)
(dAdT)_12_ **⋅**(dAdT)_12_	negligible	–	negligible	–
(dGdC)_12_ **⋅**(dGdC)_12_	(2.6±0.9)	(3.5±0.3)	(2.6±0.7)	(2.7±0.5)
22AG	(8.4±2.2)	(1.1±0.1)	(1.2±0.2)	(0.9±0.1)
c‐myc	(23.8±7.3)	(0.9±0.1)	(23.1±5.9)	(0.95±0.01)

[a] Binding site sizes are in base pairs per molecule of TAPP ([BP]/[TAPP]) for duplex DNA and in tetrads per molecule of TAPP ([tetrad]/[TAPP]) for quadruplex structures.

The binding site size for the interaction of compound **1** with duplex‐[(dGdC)_12_ **⋅**(dGdC)_12_] was determined to be in the range of three to four base pairs (Table 2 columns 2 and 4) and is in good agreement with the results from docking and calorimetric studies (see below). For the interaction of compound **1** with ct‐DNA an apparent binding site size of approximately six base pairs was observed, which is reasonable for binding displaying some extent of specificity. The fact that the apparent binding site size of six base pairs is greater than found for duplex‐[(dGdC)_12_ **⋅**(dGdC)_12_] is in line with the observation that compound **1** only binds weakly to A**⋅**T‐rich DNA. Finally, for the interaction of compound **1** with 22AG and c‐myc a ratio of three molecules of compound **1** per quadruplex, that is, one per tetrad, was observed.

#### Isothermal titration calorimetry (ITC)

Isothermal titration calorimetry was used to gain further information about the binding of compound **1** to the different duplex‐ and quadruplex‐forming DNA sequences. We first studied the ligand dilution series and observed a non‐constant heat of dilution (Figure S2 in the Supporting information), which suggests ligand self‐aggregation. Analysis of the data in terms of an isodesmic self‐aggregation model^59^ results in a good fit with an equilibrium constant for stepwise self‐aggregation, *K*_agg_, of 6.3×10^2^ m ^−1^ and an enthalpy of self‐aggregation of −4.0 kcal mol^−1^. The fact that self‐aggregation of compound **1** is exothermic is in agreement with previous observations for other (cationic) flat aromatic nucleic acid binders.^60^, ^61^, ^62^ Self‐aggregation, as quantified by *K*_agg_, is very weak for a molecule with the shape and size of compound **1**, which is unsurprising given the highly charged nature of the tetracation, and is also consistent with its excellent aqueous solubility, even in a high ionic strength buffer. This renders compound **1** a convenient nucleic acid binder with excellent potential for applications.

**Figure 3:**
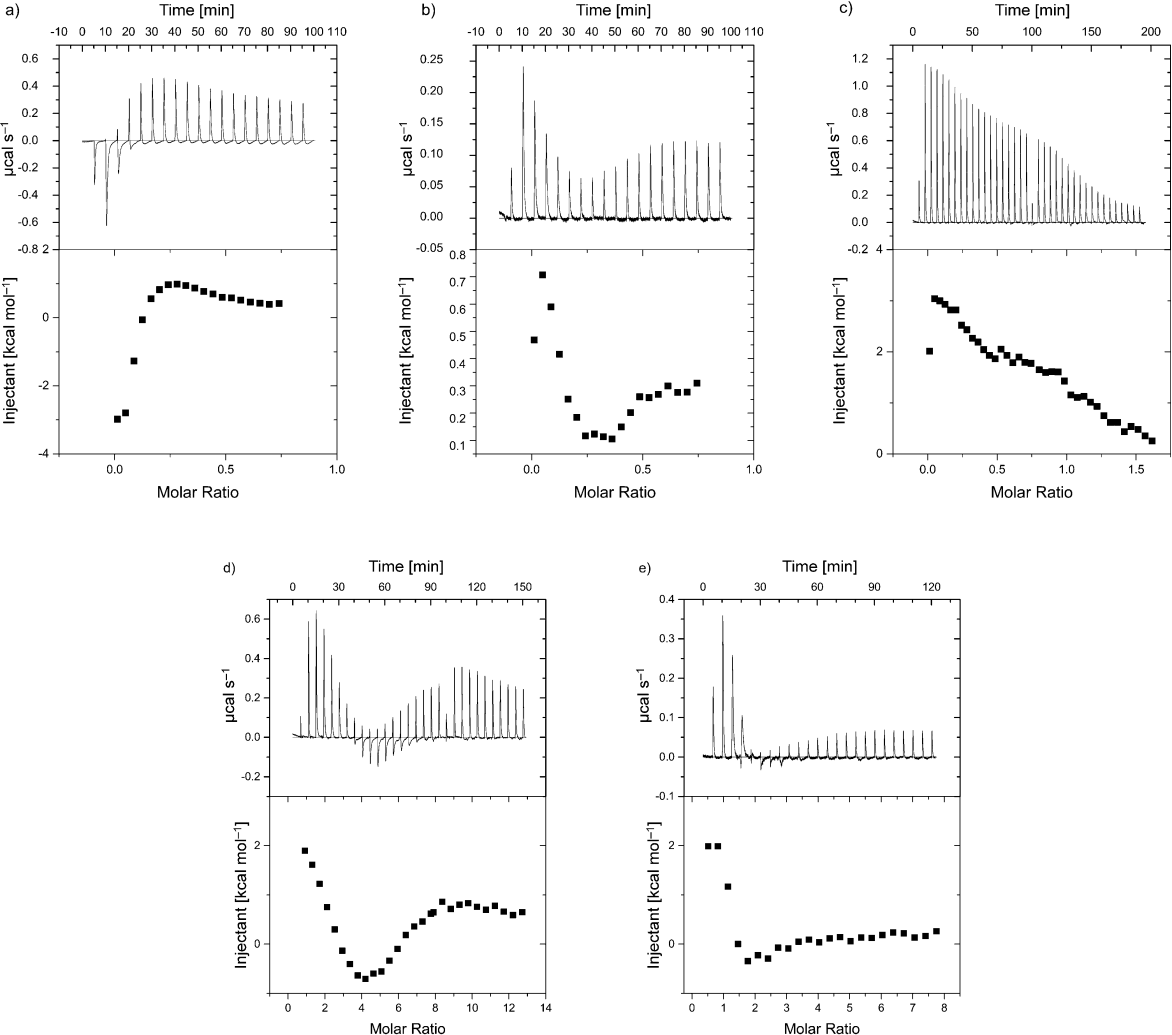
Enthalpograms for the interaction of compound **1** with a) ct‐DNA, b) duplex‐[(dGdC)_12_ **⋅**(dGdC)_12_], c) duplex‐[(dAdT)_12_ **⋅**(dAdT)_12_], d) 22AG and e) c‐myc.

**Scheme 1:**
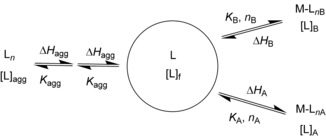
Ligand aggregation, *K*_agg_, as well as two different DNA–ligand binding events, corresponding to *K*_A_, *K*_B_, were taken into account in the analysis of the ITC data.

In the binding experiments, a solution of compound **1** was injected stepwise into solutions of the respective nucleic acid samples. Figure 3 shows the resulting enthalpograms for the interactions of compound **1** with the different sequences. In all cases, except for duplex‐[(dAdT)_12_ **⋅**(dAdT)_12_], the enthalpograms suggest one strong binding mode and weaker secondary events, followed by the heat effects for dilution of compound **1**. We therefore analysed the ITC data by using the IC ITC tool,^60^, ^63^ in terms of a model involving the nucleic acid structures having two types of binding sites in competition with ligand self‐aggregation, as illustrated in Scheme 1.

As observed in the UV/visible titrations, binding of compound **1** to duplex‐[(dAdT)_12_ **⋅**(dAdT)_12_] is too weak to be reliably analysed from the enthalpograms (Figure 3 c). On the contrary, binding of compound **1** to duplex‐[(dGdC)_12_ **⋅**(dGdC)_12_] displays two clear binding events (Figure 3 b), which is in agreement with the absence of isosbestic points in the UV/visible titration spectra. Binding model exploration through error and parameter covariance analysis^61^ (Figure S.3 a in the Supporting Information), in combination with the docking studies (and the observed tight binding of **1** to quadruplex structures through an end‐stacking binding mode, see below), led to the hypothesis that the strongest binding event corresponds to interaction with the ends of duplex‐[(dGdC)_12_ **⋅**(dGdC)_12_], whereas the second binding event corresponds to weaker interactions along the DNA strands. We therefore analysed the data restricting the first binding site to a size of twelve base pairs, that is, binding to both ends of the twenty‐four base‐pair‐long duplex. This model reproduces the data well and indicates a binding site size for the weaker interaction of 3.2 base pairs, which is in good agreement with the UV/visible titration and the docking studies. The affinity of compound **1** for duplex‐[(dGdC)_12_ **⋅**(dGdC)_12_] according to ITC is lower than derived from the spectroscopic methods, an observation, which is not uncommon for this combination of methods and has been previously reported.^60^ This apparent inconsistency between the affinities from spectroscopic and calorimetric titrations will be discussed in more detail below.

The third duplex DNA sequence, ct‐DNA (Figure 3 a), also displays multiple types of binding sites, which is readily related to its heterogeneity and also manifested in the absence of an isosbestic point in the UV/visible titration data. The observed apparent binding site sizes of 29 and seven base pairs are in agreement with the occasional presence of high affinity binding sites (possibly GC‐rich) along the DNA sequence.

The calorimetric data for compound **1** interacting with 22AG (Figure 3 d) indicate that there are two separate binding sites available on 22AG for compound **1** to interact with. Binding model exploration (Figure S3 b in the Supporting Information) shows covariance of the stoichiometries *n*_A_ and *n*_B_. Nevertheless, the best fit is observed for a stoichiometry *n*_A_ of two and *n*_B_ of four, suggesting a molecule of compound **1** binding on each side of the quadruplex in the tightest binding mode, with a weaker binding mode involving multiple molecules of compound **1**. Although the identity of the secondary binding sites is of interest, we consider attempting to identify these binding sites too speculative on the basis of our current data.

Similarly, the enthalpogram for compound **1** interacting with c‐myc displays two binding events, which again is in agreement with the absence of an isosbestic point in the UV/visible titration data. Based on binding model exploration (Figure S3 c in the Supporting Information), the calorimetrically ill‐defined *n*_B_ was restricted to two, which together with the well‐defined stoichiometry *n*_A_ of one gives a total stoichiometry of TAPP/c‐myc of 3:1, which is in agreement with the results from the spectroscopic titrations. This binding model reproduces the data well and suggests end‐stacking interactions on one side of the quadruplex stack, with the first interaction weakening the subsequent interactions at the opposite end of the quadruplex structure and with the remaining tetrad. The combined binding events thus correspond to one molecule of compound **1** per tetrad.

Table 3 summarises the binding parameters obtained from the calorimetric titrations. Overall, the binding data obtained from ITC show an affinity of compound **1** in the order c‐myc≫22AG≈ct‐DNA>duplex‐[(dGdC)_12_ **⋅**(dGdC)_12_]≫duplex‐[(dAdT)_12_ **⋅**(dAdT)_12_].

**Table 3:** Binding parameters for compound **1** interacting with different nucleic acid structures according to calorimetry.

	ct‐DNA	d(GdC)_12_ **⋅**(dGdC)_12_	22AG	c‐myc
*K*_A_ [m ^−1^]	6.18×10^6^ (1.9×10^6^–2.0×10^7^)^[a]^	1.2×10^5^ (<8×10^6^)	3.8×10^6^ (<36×10^6^)	1.3×10^7^ (>0.4×10^7^)
*n*_A_ (ligands/structure)	–	–	2.2 (1.2–4.4)	1.1 (1.0–1.3)
binding site size/base pairs (*n*_A_^−1^)	29.3 (27.8–32.2)	12^[c]^	–	–
Δ*H*_A_ [kcal mol^−1^]	−5.8 (−7.2–−5.1)	covariance with Δ*H*_B_	1.3 (<16)	1.6 (−1.4–2.0)
Δ*G*_A_ [kcal mol^−1^]	−9.3 (−10.0–−8.4)	−6.9 (>−9.4)	−9.0 (>−10.3)	−9.7 (<−9.0)
−*T*Δ*S*_A_	−3.5	n.d.^[b]^	−10.3	−11.3
*K*_B_ [m ^−1^]	3.8×10^5^ (1.1×10^5^–9.5×10^5^)	0.98×10^5^ (5.0×10^4^–7.0×10^5^)	2.3×10^5^ (0.5×10^5^–8×10^5^)	5.5×10^4^ (1.0×10^4^–6.4×10^5^)
*n*_B_	–	–	3.95 (<4.6)	2^[d]^
binding site size/base pairs (*n*_B_^−1^)	7.10 (6.5–8.1)	3.6 (2.1–6.6)	–	–
Δ*H*_B_ [kcal mol^−1^]	0.7 (0.5–1.3)	covariance with Δ*H*_A_	−2.5 (>−450)	−1.9 (>−25)
Δ*G*_b_ [kcal mol^−1^]	−7.5 (−8.2–−6.8)	−6.8 (>−7.9)	−7.3 (−8.0–−6.5)	−6.5 (>−7.9)
−*T*Δ*S*_B_	−8.2	n.d.^[b]^	−4.8	−4.6

[a] Ranges of reasonable parameter values are based on the analysis of normalised Σdev^2^/dof as a function of parameter value, see Figures S4 A and B in the Supporting Information; [b] n.d.=not determined because of covariance between Δ*H*_A_ and Δ*H*_B_; [c] restricted to a value of twelve base pairs per binding site; [d] *n*_B_ was restricted to a value of two because of being ill‐defined.

### Molecular docking studies

To gain further insight into the interaction of compound **1** and the targeted DNA sequences, molecular docking studies were carried out by using the AutoDock Vina modeling tool.^64^ For the quadruplex‐forming sequences, all available relevant structures deposited in the nucleic acids database^65^, ^66^ were used as targets. In particular for 22AG, this allowed us to compare the potential interactions of compound **1** with the parallel, antiparallel and mixed hybrid structures formed in solution. To explore the binding mode of compound **1** with duplex DNA, we used our previously developed model involving duplex DNA with a pre‐formed intercalation site as a target.^67^

**Figure 4:**
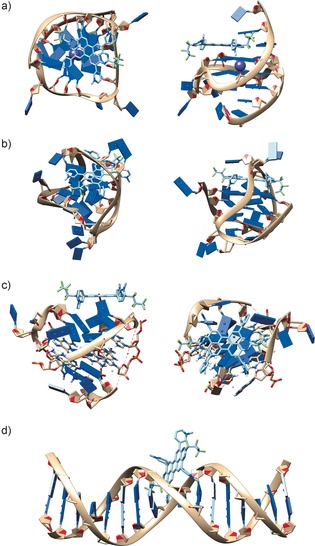
Schematic representation of the interactions of compound **1** with a) c‐myc (PDB ID: 2L7V), b) 22AG (PDB ID: 2MCO), c) the mixed‐hybrid 22AG structure (PDB ID: 2e4i) and d) duplex DNA according to molecular docking studies. Images were rendered by using UCSF Chimera.^71^

As displayed in Figure 4, the docking studies suggest that the end‐stacking binding mode, in which the planar π system of compound **1** stacks onto the external G‐quartets, is the most favourable binding mode for compound **1** interacting with c‐myc. This is in agreement with observations for other aromatic G‐quadruplex ligands.^19^, ^68^ The interaction of compound **1** with the parallel c‐myc structure from 2L7V is predicted to be favourable by −10.9 kcal mol^−1^.

The availability of the three different structures for 22AG allows us to compare the predicted binding modes and rank the predicted affinities of compound **1** for the different structures that can be formed by 22AG. From visual inspection of the top docked arrangement (Figure 4 c), the interaction of compound **1** with the mixed‐hybrid conformation of 22AG, which is relevant for our studies, appears less efficient than the interaction with c‐myc.

We note that one of the loop regions of 22AG hinders efficient stacking on top of the first quartet (Figure 4 c), though the interaction of compound **1** with 22AG is likely to involve some movement of the loop regions to allow more efficient stacking of compound **1** onto a tetrad structure. This flexibility was not represented in the docking studies. An influence of the loop regions on quadruplex recognition has recently been reported in literature.^69^, ^70^ The interaction of compound **1** with the mixed hybrid structure of 22AG is predicted to be favourable by −8.6 kcal mol^−1^, that is, significantly less than the interaction with c‐myc. This is in agreement with experiments.

Although the folding kinetics for the various 22AG structures at 25 °C suggest that the parallel and antiparallel structures of 22AG are not accessible under the reaction conditions and on the timescales relevant to this study (see above), it was of general interest to explore the effect of the different structures on the affinity of compound **1** for 22AG. The interactions of compound **1** with the antiparallel 22AG structure from 2MCO and the parallel 22AG structure from 1KF1 (not shown) are predicted to be favourable by −12.5 and −11.7 kcal mol^−1^, respectively. The predicted interaction with the parallel c‐myc structure and the parallel structure for 22AG (which is inaccessible under our experimental conditions) are similar. Docking studies thus suggest that the difference in affinity of compound **1** for c‐myc and 22AG may be related to the fact that only the mixed hybrid structure of 22AG is available under our experimental conditions whereas compound **1** has a higher affinity for parallel quadruplexes. However, this higher affinity cannot drive a change in the structure to the parallel structure on the timescale of our experiments because of the slow folding kinetics of 22AG. This is in agreement with the observation that the circular dichroism spectrum of 22AG does not change in the presence of compound **1**. Docking studies are thus in agreement with the hypothesis that compound **1** distinguishes between the parallel and mixed‐hybrid conformations.

Finally, the docking studies for compound **1** interacting with duplex DNA (Figure 4 d) suggest that compound **1** does not enter the pre‐formed intercalation gap but binds in the minor groove instead. This interaction mode is rather inefficient compared to the π‐stacking mode observed for the quadruplex structures, providing a rationale for the higher affinity of compound **1** for G‐quadruplex over duplex DNA. A likely reason for the non‐intercalative binding is the size of the molecule, which does not allow compound **1** to thread through the DNA double helix.

### Discussion of the affinities and binding modes according to the spectroscopic, calorimetric and docking studies

For some of the DNA sequences investigated in this work there appears to be an inconsistency between the affinities and binding modes derived from the spectroscopic, calorimetric and docking studies. This is largely due to the fact that the spectroscopic data were analysed in terms of a multiple independent binding site model, involving one type of binding site. On the other hand, the calorimetric data was subjected to an analysis in terms of a model involving two types of binding sites. The binding parameters derived from these two types of data analyses have previously been shown not to be directly comparable,^60^ but may be related through analysis of simulated data as detailed in the Supporting Information.

First of all, both the spectroscopic and calorimetric titrations confirm that the interactions between compound **1** and with duplex‐[(dAdT)_12_ **⋅**(dAdT)_12_] are weak and we have not studied these in further detail. On the other hand, the optimised parameters from the spectroscopic studies of the interactions of compound **1** with duplex‐[(dGdC)_12_ **⋅**(dGdC)_12_], (Table 2) appear to be inconsistent with those from the calorimetric studies (Table 3).

As detailed in Section S2 of the Supporting Information, there is a considerable range of acceptable values for several of the parameters obtained from the analysis (in terms of two inequivalent types of binding sites) of the calorimetric data. Comparison of the concentration profiles predicted by reasonable calorimetric models shows that several of these are in very good agreement with those obtained from the spectroscopic titrations (Table S2 in the Supporting Information). Based on this combined analysis of the spectroscopic and calorimetric data, a value for *K*_A_ of around 5×10^6^ m ^−1^, a value for *K*_B_ of around 5×10^5^ m ^−1^ and a binding site size *n*_B_^−1^ of 2.5 base pairs rather than the optimised values based on the calorimetric titrations alone may better represent the ligand–DNA interaction in the case at hand. The resulting binding site size is also in reasonable agreement with the docking studies.

The binding parameters for 22AG, which also seemed to differ slightly based on the chosen method (see above) were subjected to a comparison analogous to that used for duplex‐[(dGdC)_12_ **⋅**(dGdC)_12_] (Section S2 in the Supporting Information). Here, comparison of the concentration profiles suggests that the calorimetric model involving two high affinity binding sites and four lower affinity binding sites per quadruplex structure is in fact in good agreement with the results from the spectroscopic titrations (Table S2 in the Supporting Information). The two high affinity binding sites agree with the docking studies. However, the nature of the additional low affinity binding sites remains unclear.

We have not attempted to similarly consolidate the interaction parameters for compound **1** interacting with ct‐DNA because the heterogeneity of ct‐DNA, resulting in a range of binding site types, makes detailed comparison of models involving only one and two types of sites meaningless.

Finally, the high affinity of compound **1** for the parallel c‐myc structure is confirmed by both spectroscopic and calorimetric experiments. The affinity according to the spectroscopic titrations is in good agreement with the *K*_A_ value from the calorimetric titrations, possibly because the secondary binding sites are considerably weaker than the highest affinity binding sites in this system. The total stoichiometry of three molecules of compound **1** per quadruplex is confirmed by both types of titrations. The docking studies involved a rigid c‐myc target and the total stoichiometry of three could therefore not be confirmed through our docking studies.

## Conclusion

The water‐soluble tetraazaperopyrene dye **1** displays a remarkably high affinity of 2×10^7^ m ^−1^ (i.e., *K*_d_=50 nm) for the c‐myc quadruplex structure, which places this structure amongst the strongest quadruplex binders currently known.^33^, ^39^ Moreover, compound **1** displays a preference for binding to c‐myc not only in relation to duplex‐forming nucleic acid structures, but also relative to the alternative quadruplex‐forming structure 22AG in its mixed‐hybrid structure. In light of the low relative abundance of quadruplex structures relative to duplex structures in vivo the observed selectivity is not high enough yet for bioimaging purposes. Applications in directed assembly of functional nanostructures, however, do not suffer from significant differences in concentrations of duplex and quadruplex DNA and are viable already. In addition, compound **1** provides a new structural class of quadruplex binders and no efforts were made thus far to increase the selectivity. In fact, the current study highlights the potential of the molecular scaffold of compound **1** in quadruplex recognition and allows the identification of structural variation to improve the structural selectivity of this class of compounds. Similarly, direct competition studies, involving the competition dialysis assay of a wider range of nucleic acid structures are ongoing. Together with our previous observations that compound **1** is a nucleus‐selective stain,^47^ its excellent water solubility and spectroscopic properties, the preference for quadruplex structures and c‐myc in particular makes TAPP derivative **1** a highly promising agent for selectively addressing the c‐myc promotor sequence, be it for therapeutic or biosensing applications, or in directed assembly.

## Experimental Section

**General remarks**: UV/visible spectra were recorded by using a Jasco 630 or 650 UV/visible spectrophotometer equipped with a Peltier temperature controller. The pH values of aqueous solutions were determined by using a Hanna Instruments pH210 microprocessor pH meter with a VWR simple junction gel universal combined pH/reference electrode.

**DNA binding experiments**: Water was purified by using a Purelab Option R7 water purifier. MOPS (3‐(*N*‐morpholino)propanesulfonic acid, CAS [1132‐61‐2]), NaCl and EDTA (ethylenediaminetetraacetic acid disodium salt dihydrate, CAS [6381‐92‐6]) were obtained from Fisher and used as supplied. Oligonucleotides were obtained from custom synthesis (Yorkshire Bioscience), dissolved in buffer and dialysed (3.5 kDa MWCO, Visking, Medicell International Ltd) extensively against two litres of buffer. All experiments were carried out in aqueous MOPS buffers (25 mm MOPS titrated to pH 7.1, 1 mm EDTA and 100 mm KCl). Dialysed solutions of oligonucleotides were quantified by spectrophotometry by using:

*ɛ*_260 nm, 22AG_=228 500 m ^−1^ (quadruplex) cm^−1^ (22AG=dAdGd‐GdGdTdTdAdGdGdGdTdTdAdGdGdGdTdTdAdGdGdG)^72^

*ɛ*_260 nm, c‐myc_=228 700 m ^−1^ (quadruplex) cm^−1^ (c‐myc=dTd‐GdAdGdGdGdTdGdGdGdTdAdGdGdGdTdGdGdGdTdAdA)^66^

*ɛ* ${}_{262\;\mathrm{nm},\;\left(\mathrm{dAdT}\right){}_{12}\cdot \left(\mathrm{dAdT}\right){}_{12}}$ =13 200 m ^−1^ (base pairs) cm^−1[73]^

*ɛ* ${}_{254\;\mathrm{nm},\;\left(\mathrm{dGdC}\right){}_{12}\cdot \left(\mathrm{dGdC}\right){}_{12}}$ =16 800 m ^−1^ (base pairs) cm^−1[73]^

*ɛ*_2260 nm, ct‐DNA_=12 800 m ^−1^ (base pairs) cm^−1[67]^

The extinction coefficients for the quadruplex‐forming sequences were not corrected for folding‐induced hypochromism, which has been reported to be small.^74^ All solutions were annealed by heating to 95 °C for at least 5 min followed by cooling slowly to room temperature.

Fluorescence spectra were measured with a Varian Cary Eclipse spectrophotometer, the cuvettes were held at constant temperature of 20 °C and standard corrections were applied to all spectra. The excitation wavelength for all experiments was *λ*=460 nm.

**Isothermal titration calorimetry (ITC)**: Calorimetric titrations were carried out at 25 °C on a high‐precision VP‐ITC microcalorimeter (MicroCal, LLC Northampton, MA).^75^ The instrument was operated in high gain mode, applying a reference power of 10 μcal s^−1^ while stirring the sample cell contents at 307 rpm. Concentrated solutions of the nucleic acids were dialysed (MWCO 3.5 kDa) extensively against buffer, and diluted by using the final dialyzate to concentrations as required. Ligand solutions were freshly prepared by using the final nucleic acid dialyzates, with a typical ligand concentration of 0.75 mm. All solutions were degassed immediately before use. Typically, ligand dilution experiments were set up so that 15 μL of ligand solution were added to a known volume (approximately 1.9 mL including overflow) of buffer in the sample cell every 5 min up to a total of 20 injections. Titrations involving nucleic acids typically involved a 15 μL injection once every 5 min. Titrations were concatenated if required. The raw data were treated as usual by using Origin to generate both integrated heat effects per injection (dh) and molar heat effects per injection (ndh). The integrated heat data were subsequently analysed by using IC ITC.^60^, ^63^

**Docking studies**: Docking studies were carried out by using the Autodock Vina 1.1.2 modelling tool.^64^ The required PDBQT files for TAPP **1** and for the quadruplex structures were generated by using AutoDockTools 1.5.6 Sep 17 14.^76^, ^77^ A crystal structure^47^ was used for TAPP **1**. Quadruplex structures were selected from the nucleic acid database^65^, ^66^ (see the Supporting Information for selection criteria). The grid box dimensions for the docking studies involving quadruplex structures were 40 Å×40 Å×40 Å (determined by visual inspection so that the grid box encompassed the quadruplex structures, but also provided additional space to allow for maximum flexibility in ligand orientations). The construction of the PDBQT file and the grid box dimensions for the rigid target duplex DNA structure displaying a pre‐formed intercalation gap were described previously.^67^ The nucleic acid structures were kept rigid in the docking studies and polar hydrogen atoms were added. Docked poses were visualised by using UCSF Chimera.^71^

## Supporting information

As a service to our authors and readers, this journal provides supporting information supplied by the authors. Such materials are peer reviewed and may be re‐organized for online delivery, but are not copy‐edited or typeset. Technical support issues arising from supporting information (other than missing files) should be addressed to the authors.

Supplementary
