# Supplementary material for: A Water‐Soluble Tetraazaperopyrene Dye as Strong G‐Quadruplex DNA Binder
Source: Chemistry. 2016 Mar 21;22(18):6314–22. doi: 10.1002/chem.201504934 (PMC5071672; doi:10.1002/chem.201504934)
Supplement: Supplementary file 1 — Supplementary [file CHEM-22-6314-s001.pdf]

CHEMISTRY
A European Journal

Supporting Information

A Water-Soluble Tetraazaperopyrene Dye as Strong G-Quadruplex DNA Binder

Lena Hahn,[a] Niklaas J. Buurma,*[b] and Lutz H. Gade*[a]

chem_201504934_sm_miscellaneous_information.pdf

Supporting Information

A Water-soluble Tetraazaperopyrene Dye as Strong G-Quadruplex DNA Binder

Lena Hahn, Niklaas J. Buurma* and Lutz H. Gade *

TOC

Equation S1	SI-2
Figures S1a	SI-3
Figures S1b	SI-5
Figure S2	SI-7
Figures S3a-c	SI-8
Figures S4a-d	SI-9
S1 Docking studies – selection criteria and treatment of structures	SI-18
S2 Comparison of interaction parameters from spectroscopic and calorimetric studies	SI-19
Figure S5a/b	S-22
S3 References	SI-23

[a] Dr. L. Hahn, Prof. Dr. L. H. Gade
Anorganisch-Chemisches-Institut, Universität Heidelberg
Im Neuenheimer Feld 270, 69120 Heidelberg (Germany)
Fax: (+49) 6221545609
E-mail: lutz.gade@uni-hd.de

[b] Dr. N. J. Buurma
Physical Organic Chemistry Centre, School of Chemistry, Cardiff University, Main Building
Park Place, Cardiff, CF10 3AT, United Kingdom
Fax: (+44) (0)2920874030

Supporting information for this article is given via a link at the end of the document.

Equation S1

$$signal_{\text{obsd}} = signal_{\text{background}} + signal_{\text{free,m}} \cdot [\text{L}]_{\text{tot}} + \Delta_{\text{binding}} signal_{\text{m}} \cdot \left\{ \frac{1 + K \cdot \frac{[\text{DNA}]_{\text{tot}}}{n} + K \cdot [\text{L}]_{\text{tot}} - \sqrt{\left(1 + K \cdot \frac{[\text{DNA}]_{\text{tot}}}{n} + K \cdot [\text{L}]_{\text{tot}}\right)^2 - 4 \cdot K^2 \cdot \frac{[\text{DNA}]_{\text{tot}}}{n} \cdot [\text{L}]_{\text{tot}}}}{2 \cdot K} \right\}$$

parameter		Description
$signal_{\text{obsd}}$		observed signal (absorption for UV/visible spectroscopy)
$signal_{\text{background}}$		background signal (baseline UV/visible absorption of buffer and cuvette for UV/visible spectroscopy)
$signal_{\text{free,m}}$		molar signal of the free ligand (extinction coefficient for UV/visible spectroscopy)
$[\text{L}]_{\text{tot}}$		total ligand concentration
$\Delta_{\text{binding}} signal_{\text{m}}$		change in the molar signal upon binding (change in the extinction coefficient for UV/visible spectroscopy)
K		equilibrium constant
$[\text{DNA}]_{\text{tot}}$		concentration of added DNA
N		binding site size (defined as concentration of binding sites added = $[\text{DNA}]_{\text{tot}}$ / n)

Derivation

Equation S1 is valid for titrations carried out using spectroscopic detection methods (UV-visible, fluorescence and/or circular dichroism spectroscopy) at wavelengths where only the nucleic acid binder causes a signal. The observed signal ($signal_{\text{obsd}}$) is then the result of the concentrations of the free and bound nucleic acid binder ($[\text{L}]_{\text{free}}$ and $[\text{L}]_{\text{bound}}$) and their respective molar response factors ($signal_{\text{free,m}}$ and $signal_{\text{bound,m}}$) in combination with any background signal ($signal_{\text{background}}$). Defining the change in the molar response factors as $\Delta_{\text{binding}} signal_{\text{m}}$ = $signal_{\text{bound,m}}$ - $signal_{\text{free,m}}$ and the total ligand concentration as $[\text{L}]_{\text{total}}$ = $[\text{L}]_{\text{free}}$ + $[\text{L}]_{\text{bound}}$ then gives:

$$signal_{\text{obsd}} = signal_{\text{background}} + signal_{\text{free,m}} \cdot [\text{L}]_{\text{free}} \cdot l + signal_{\text{bound,m}} \cdot [\text{L}]_{\text{bound}} \cdot l \therefore$$
$$signal_{\text{obsd}} = signal_{\text{background}} + signal_{\text{free,m}} \cdot [\text{L}]_{\text{free}} \cdot l + \{signal_{\text{free,m}} - \Delta_{\text{binding}} signal_{\text{m}}\} \cdot [\text{L}]_{\text{bound}} \cdot l \therefore$$
$$signal_{\text{obsd}} = signal_{\text{background}} + signal_{\text{free,m}} \cdot [\text{L}]_{\text{free}} \cdot l + signal_{\text{free,m}} \cdot [\text{L}]_{\text{bound}} \cdot l - \Delta_{\text{binding}} signal_{\text{m}} \cdot [\text{L}]_{\text{bound}} \cdot l \therefore$$
$$signal_{\text{obsd}} = signal_{\text{background}} + signal_{\text{free,m}} \cdot [\text{L}]_{\text{tot}} \cdot l + \Delta_{\text{binding}} signal_{\text{m}} \cdot [\text{L}]_{\text{bound}} \cdot l$$

For every data point, $[\text{L}]_{\text{tot}}$ and the total DNA concentration, $[\text{DNA}]_{\text{tot}}$, are known. We therefore require $[\text{L}]_{\text{bound}}$ to be expressed in terms of $[\text{L}]_{\text{tot}}$ and $[\text{DNA}]_{\text{tot}}$.

The binding equilibrium is most conveniently defined in terms of concentrations of binding sites, rather than in terms of concentrations of DNA basepairs.

For a binding site size of *n* basepairs, i.e. one ligand binds to *n* basepairs, the total concentration of binding sites in solution is given by:

$[\text{binding sites}]_{\text{tot}}$ = $[\text{DNA}]_{\text{tot}}$ / n

The binding equilibrium is defined as:

$$\text{free binding sites} + \text{free ligand} \rightleftharpoons \text{complex}$$

Where the concentration of the complex, [complex], is the concentration of bound ligand $[L]_{bound}$, which equals the concentration of occupied (bound) binding sites $[\text{binding sites}]_{bound}$. This gives the equilibrium constant as

$$[\mathrm{L}]_{\mathrm{bound}} = K \cdot [\mathrm{L}]_{\mathrm{free}} \cdot [\text{binding sites}]_{\mathrm{free}}$$

In addition:

$$[\mathrm{L}]_{\mathrm{free}} = [\mathrm{L}]_{\mathrm{tot}} - [\mathrm{L}]_{\mathrm{bound}}$$
$$[\text{binding sites}]_{\mathrm{free}} = [\text{binding sites}]_{\mathrm{tot}} - [\text{binding sites}]_{\mathrm{bound}} = [\text{binding sites}]_{\mathrm{tot}} - [\mathrm{L}]_{\mathrm{bound}}$$

Therefore:

$$[\mathrm{L}]_{\mathrm{bound}} = K \cdot \{[\mathrm{L}]_{\mathrm{tot}} - [\mathrm{L}]_{\mathrm{bound}}\} \cdot \{[\text{binding sites}]_{\mathrm{tot}} - [\mathrm{L}]_{\mathrm{bound}}\} \therefore$$
$$[\mathrm{L}]_{\mathrm{bound}} = K \cdot [\mathrm{L}]_{\mathrm{tot}} \cdot [\text{binding sites}]_{\mathrm{tot}} - K \cdot [\mathrm{L}]_{\mathrm{tot}} \cdot [\mathrm{L}]_{\mathrm{bound}} - K \cdot [\text{binding sites}]_{\mathrm{tot}} \cdot [\mathrm{L}]_{\mathrm{bound}} + K \cdot [\mathrm{L}]^2_{\mathrm{bound}} \therefore$$
$$0 = K \cdot [\mathrm{L}]^2_{\mathrm{bound}} - \{1 + K \cdot [\mathrm{L}]_{\mathrm{tot}} + K \cdot [\text{binding sites}]_{\mathrm{tot}}\} \cdot [\mathrm{L}]_{\mathrm{bound}} + K \cdot [\mathrm{L}]_{\mathrm{tot}} \cdot [\text{binding sites}]_{\mathrm{tot}}$$

The quadratic equation is solved for $[L]_{bound}$ in the usual manner for quadratic equations. Inspection of the two solutions shows that only

$$[\mathrm{L}]_{\mathrm{bound}} = \left\{ \frac{1 + K \cdot [\text{binding sites}]_{\mathrm{tot}} + K \cdot [\mathrm{L}]_{\mathrm{tot}} - \sqrt{(1 + K \cdot [\text{binding sites}]_{\mathrm{tot}} + K \cdot [\mathrm{L}]_{\mathrm{tot}})^2 - 4 \cdot K^2 \cdot [\text{binding sites}]_{\mathrm{tot}} \cdot [\mathrm{L}]_{\mathrm{tot}}}}{2 \cdot K} \right\}$$

corresponds to a physically reasonable solution.

Back-substitution of $[\text{binding sites}]_{tot} = [DNA]_{tot} / n$ then gives

$$[\mathrm{L}]_{\mathrm{bound}} = \left\{ \frac{1 + K \cdot \frac{[\mathrm{DNA}]_{\mathrm{tot}}}{n} + K \cdot [\mathrm{L}]_{\mathrm{tot}} - \sqrt{\left(1 + K \cdot \frac{[\mathrm{DNA}]_{\mathrm{tot}}}{n} + K \cdot [\mathrm{L}]_{\mathrm{tot}}\right)^2 - 4 \cdot K^2 \cdot \frac{[\mathrm{DNA}]_{\mathrm{tot}}}{n} \cdot [\mathrm{L}]_{\mathrm{tot}}}}{2 \cdot K} \right\}$$

which combines with

$$signal_{\mathrm{obsd}} = signal_{\mathrm{background}} + signal_{\mathrm{free,m}} \cdot [\mathrm{L}]_{\mathrm{tot}} \cdot l + \Delta_{\mathrm{binding}} signal_{\mathrm{m}} \cdot [\mathrm{L}]_{\mathrm{bound}} \cdot l$$

to give Equation S1.

Equation S1 reproduces the observed signal as a function of $[DNA]_{tot}$ and $[L]_{tot}$. and can therefore be fitted to data of the general form for every data point *i*:

$[DNA]_{tot,i}$, $[L]_{tot,i}$, $signal_i$

The use of actual calculated $[L]_{tot,i}$ for every data point *i* allows us to correct for ligand dilution during the titration.

Figure S1a: Fluorescence spectra

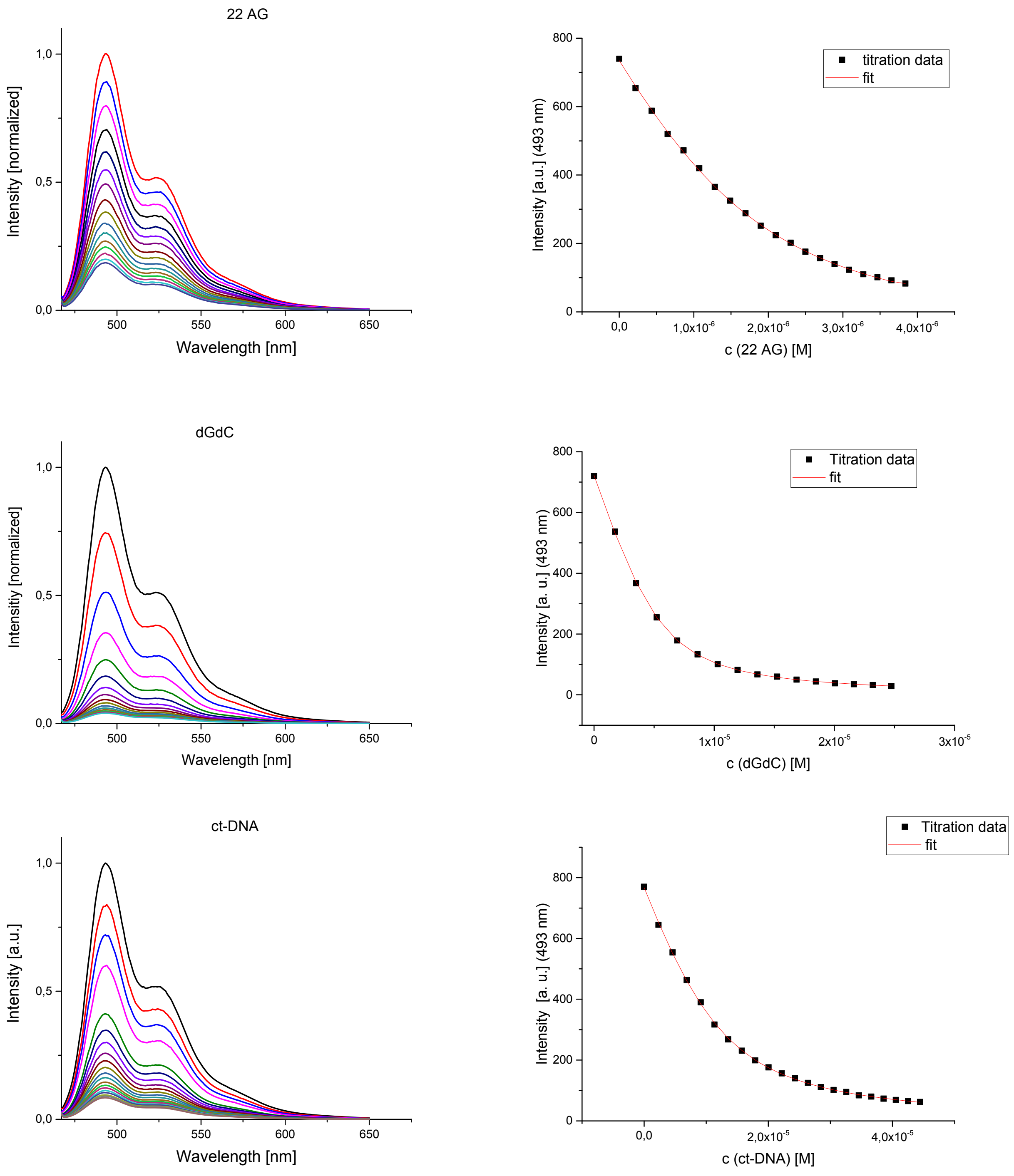

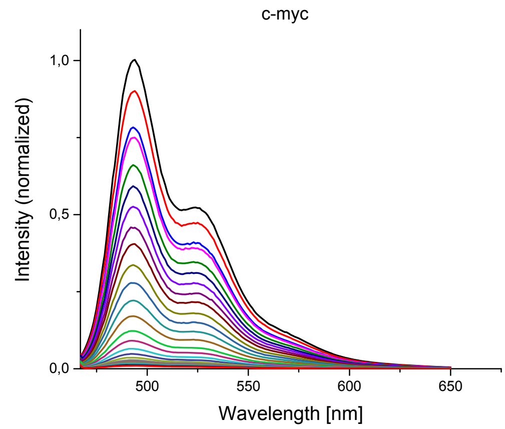
c-myc
Intensity (normalized)
1,0
0,5
0,0
500
550
600
650
Wavelength [nm]

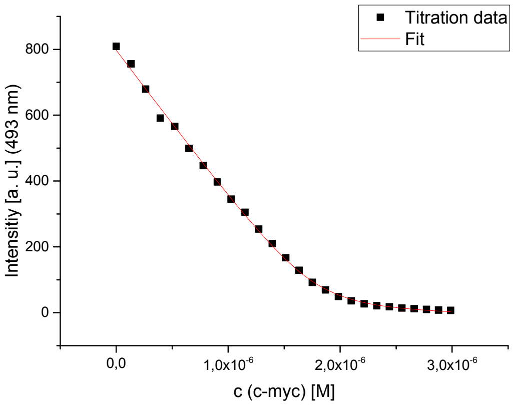
Titration data
Fit
Intensitiy [a. u.] (493 nm)
800
600
400
200
0
0,0
1,0x10^{-6}
2,0x10^{-6}
3,0x10^{-6}
c (c-myc) [M]

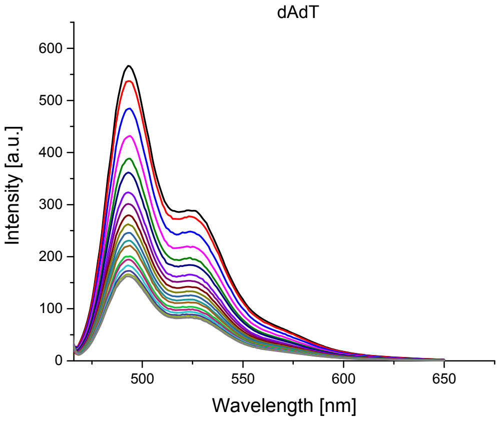
dAdT
Intensity [a.u.]
600
500
400
300
200
100
0
500
550
600
650
Wavelength [nm]

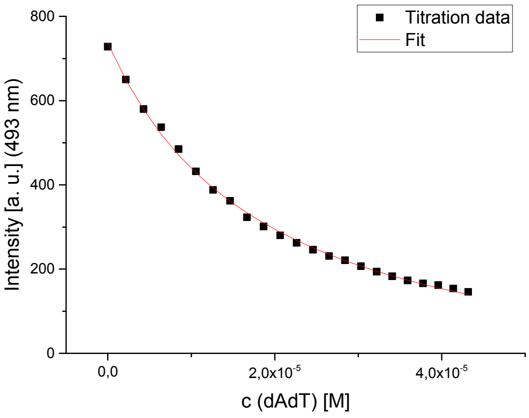
Titration data
Fit
Intensity [a. u.] (493 nm)
800
600
400
200
0
0,0
2,0x10^{-5}
4,0x10^{-5}
c (dAdT) [M]

Figure S1b: Fluorescence titration up to 2 equivalents of each DNA sequence.

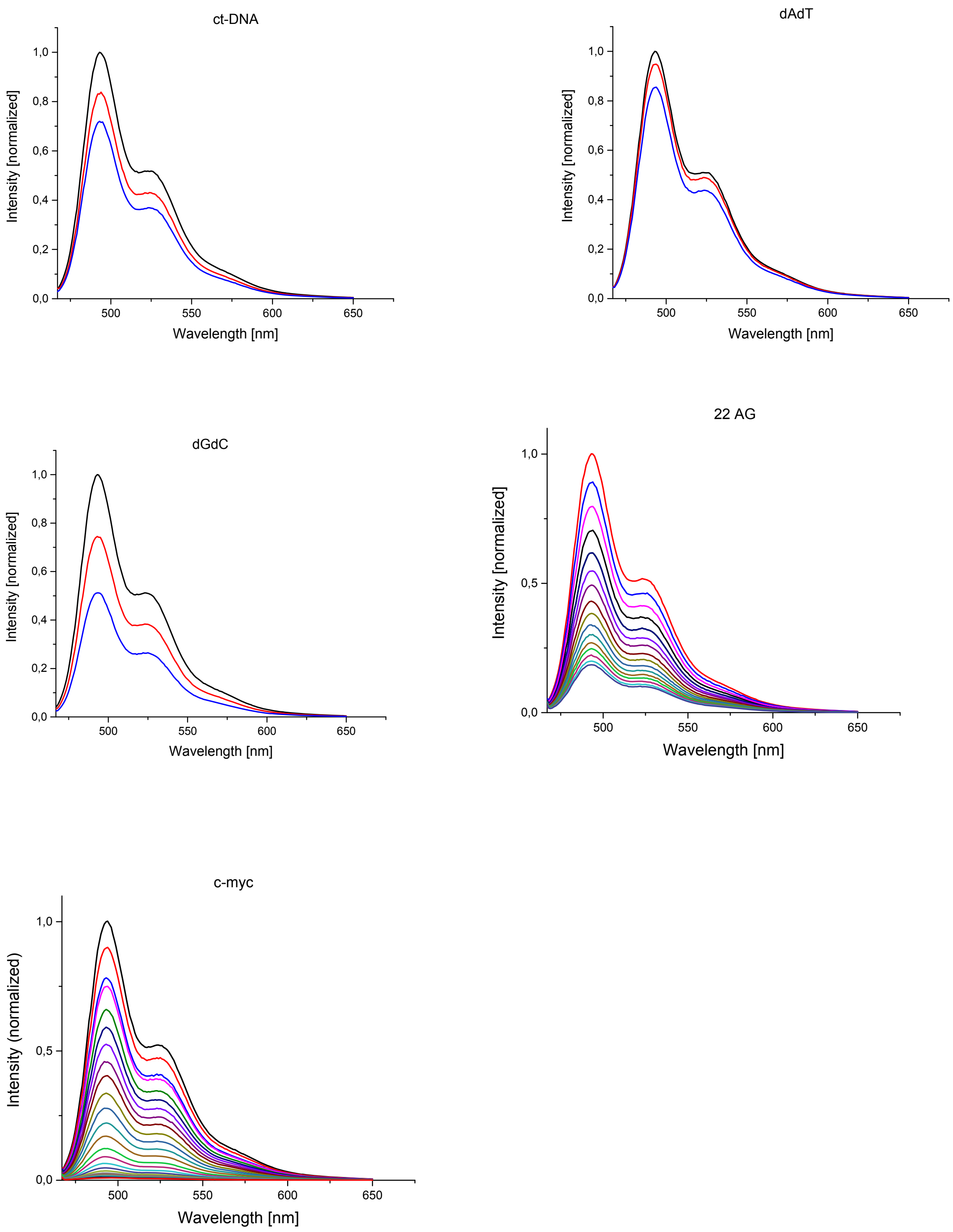

Figure S1c: UV/Vis-spectra

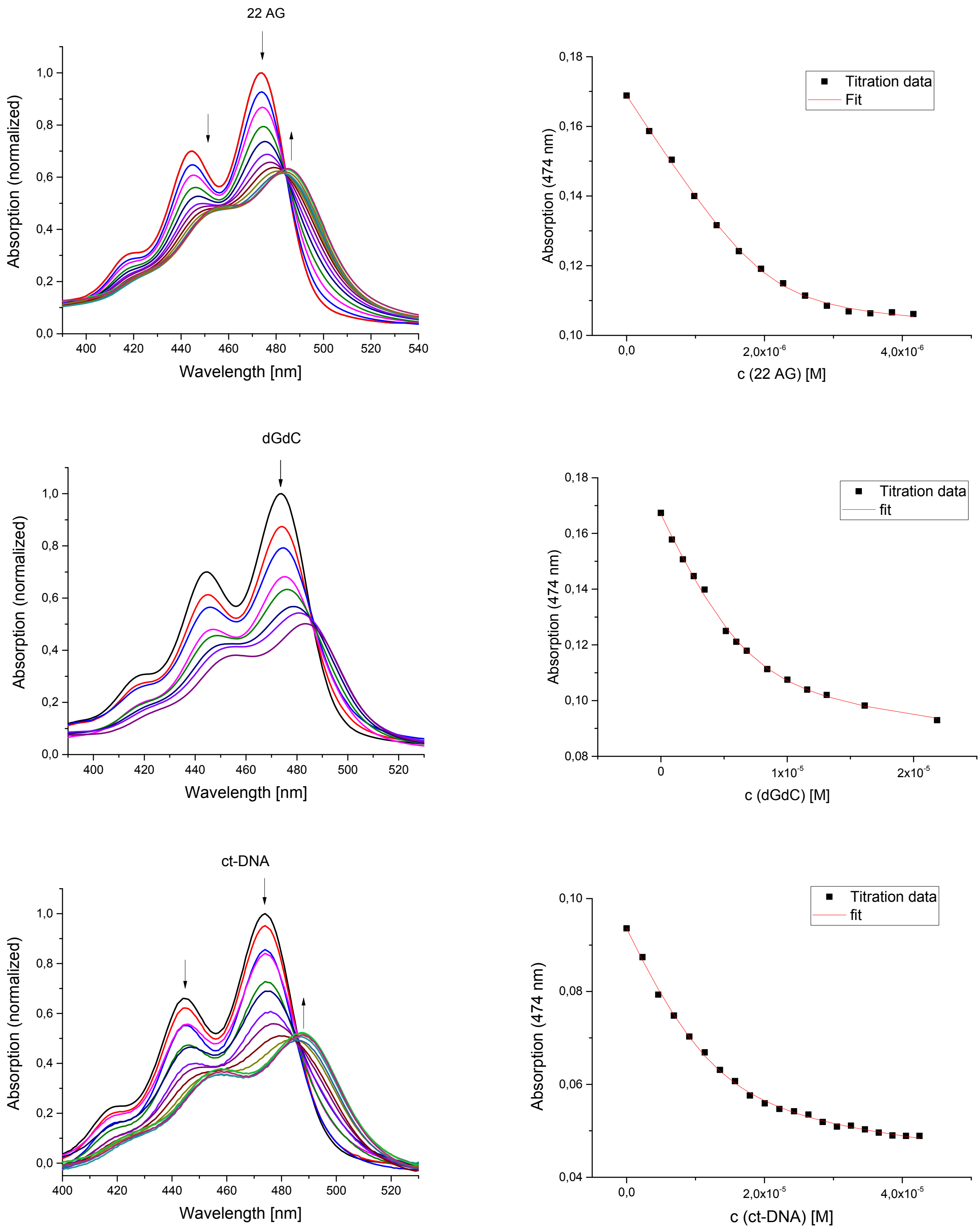

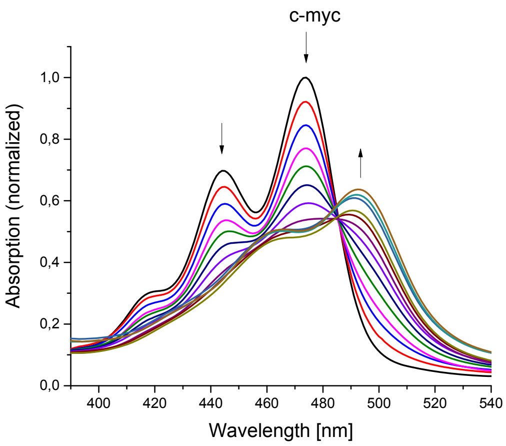
c-myc
Absorption (normalized)
Wavelength [nm]

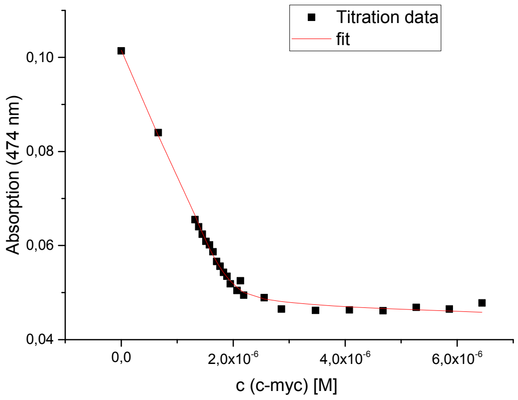
Titration data
fit
Absorption (474 nm)
c (c-myc) [M]

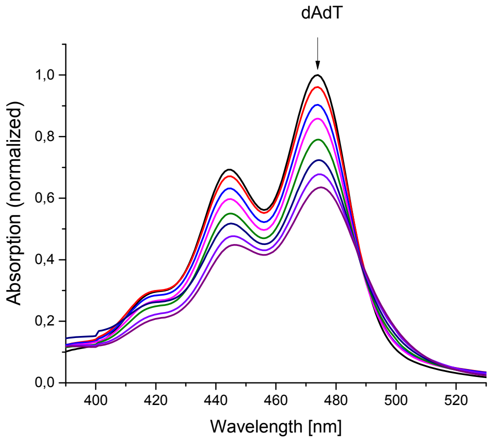
dAdT
Absorption (normalized)
Wavelength [nm]

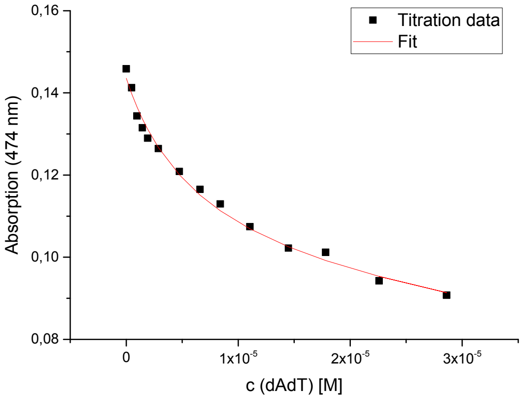
Titration data
Fit
Absorption (474 nm)
c (dAdT) [M]

Figure S2: Dilution Experiments

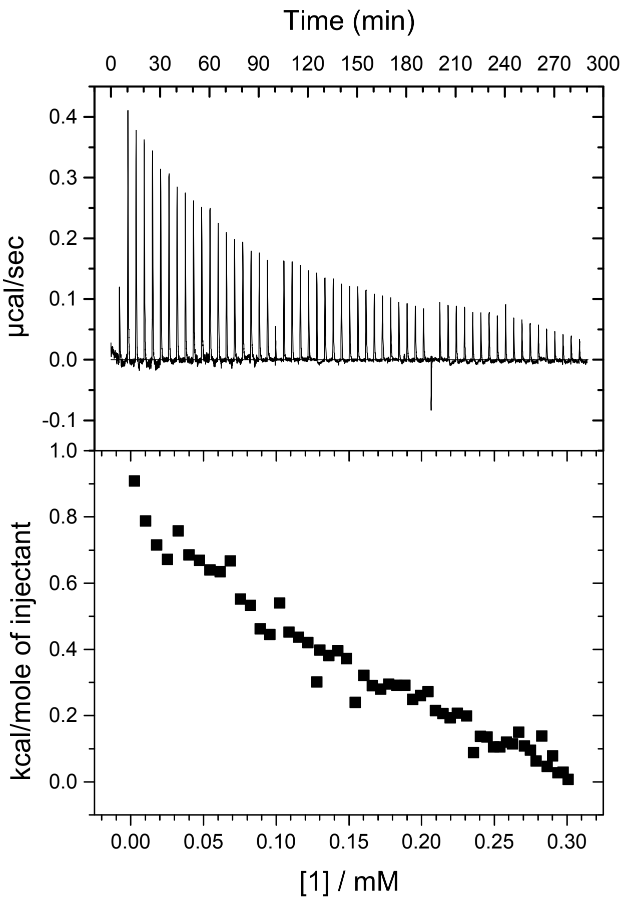

Heat effects for dilution of 0.75 mM **1** in 25 mM MOPS, 100 mM KCl, 1 mM EDTA, pH 7.1 at 25 °C.

Figure S3: Binding model exploration

The potential binding models were explored by fitting a model involving ligand aggregation, as quantified by the independently determined K_{agg} and ΔH_{agg}, as well as the two different DNA-ligand binding events to the calorimetric data. For the two different binding events, K_A, ΔH_{agg}, n_A, K_B, ΔH_{agg} and n_B were all optimised without restrictions using the simulated annealing protocols incorporated in I2CITC. Following fitting, the simulated annealing trajectories were analysed to obtain plots of the normalised sum over square deviations divided by degrees of freedom ($\Sigma dev^2/dof$) as a function of the stoichiometries n_A and n_B with all other parameters as shown in Scheme 1 having their optimised values for each particular ombination of n_A and n_B. (Figures S3A-C). Figures S3A-C thus show the lowest value of $\Sigma dev^2/dof$ for each combination of n_A and n_B. Combinations of parameter values for which normalised $\Sigma dev^2/dof$ is less than 2 should be considered within error margins. For further details, see reference [1].

**A**<br><br>**dGdC**	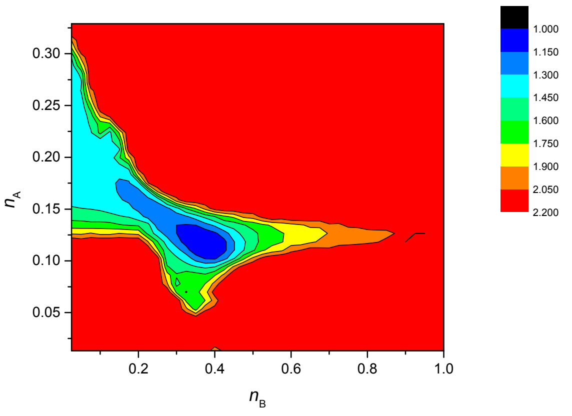	Although higher values for n_A are possible in combination with very small values of n_B, the best fits are for relatively low values of n_A from just below 0.10 up to approximately 0.14 ligands per base pair. This corresponds to relatively large binding site sizes of seven to over ten basepairs. An interpretation of an extended binding site size of 12 base pairs (n_A = 0.083) corresponds to binding to both ends of the 24 base-pair-long duplex. The value of n_A was therefore restricted to 0.083 in subsequent fits.
**B**<br><br>**22AG**	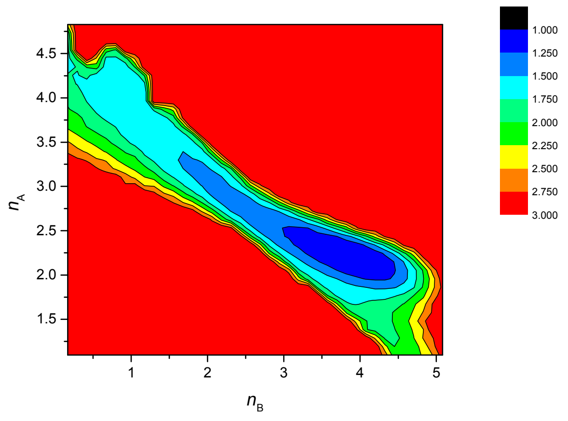	There is covariance of the stoichiometries n_A and n_B. Nevertheless, the best fit is observed for a stoichiometry n_A of two and n_B of four, suggesting a molecule of **1** binding on each side of the quadruplex in the tightest binding mode, with a weaker binding mode involving multiple molecules of **1**. No parameters were restricted in the fitting.

**C**<br><br>**c-myc**	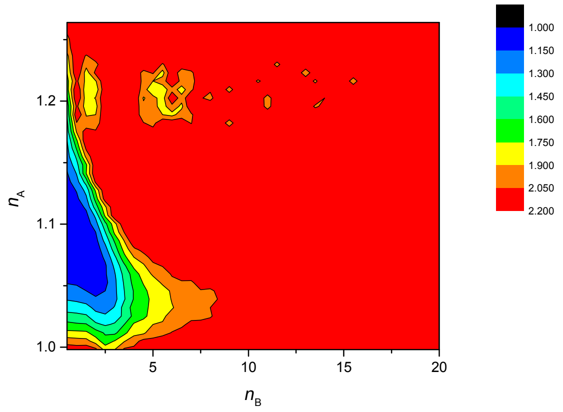 	The value of n_A is well defined as approximately 1. The parameter n_B is calorimetrically ill-defined with values up to at least 5 within error margins. n_B was therefore restricted to a value of two, which, together with the well-defined stoichiometry n_A of one, gives a total stoichiometry TAPP : c-myc of 3:1, in agreement with the results from the spectroscopic titrations.

Figure S4: Fits and evaluation of error margins and parameter covariance

The error margins on the optimised parameters for calorimetric data for 1 binding to the various nucleic acid structures were evaluated in a manner analogous to the exploration of the binding models above. Following fitting, the simulated annealing trajectories were analysed to obtain plots of the normalised sum over square deviations divided by degrees of freedom ($\Sigma dev^2/dof$) as a function of each parameter, with all other optimisable parameters having their optimised values for each particular value of the parameter of interest (Figures S4A-D). Figures S4A-D thus show the lowest value of $\Sigma dev^2/dof$ for value of a particular parameter. Parameter values for which normalised $\Sigma dev^2/dof$ is less than 2 should be considered within error margins. Figure S4.1 illustrates how error margins are determined for the binding site size n_A for the strongest binding mode of **1** with ct-DNA.

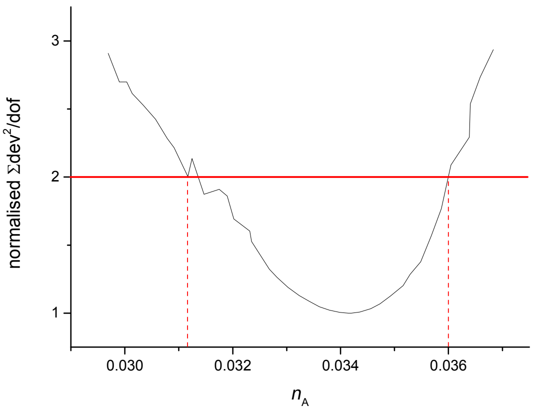

Figure S4.1: Normalised sum over square deviations divided by degrees of freedom, normalised ($\Sigma dev^2/dof$), as a function of n_A with all other parameter optimised.

Figure S4.1 shows the normalised sum over square deviations divided by degrees of freedom, normalised ($\Sigma dev^2/dof$), as a function of n_A with all other parameters at the values giving the best possible fit for the given value of n_A. According to Figure 1, the lowest value of n_A giving an acceptable fit corresponds to 0.0311 ligands per basepair whereas the highest value of n_A giving a reasonable fit is 0.0360 ligands per basepair. This corresponds to the range of reasonable binding sizes being 27.8 – 32.2.

For further details, see reference [1].

A: ct-DNA

Fitted data

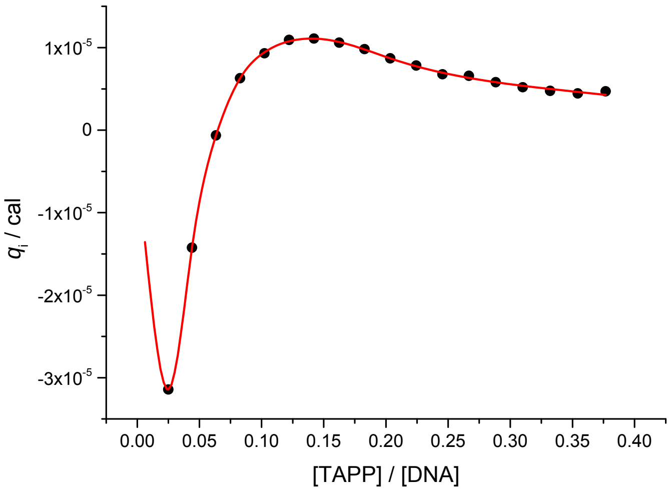

Normalised Σdev^2/dof as a function of optimisable parameter values; parameter values for which the normalised Σdev^2/dof is less than 2 should be considered within error margins.

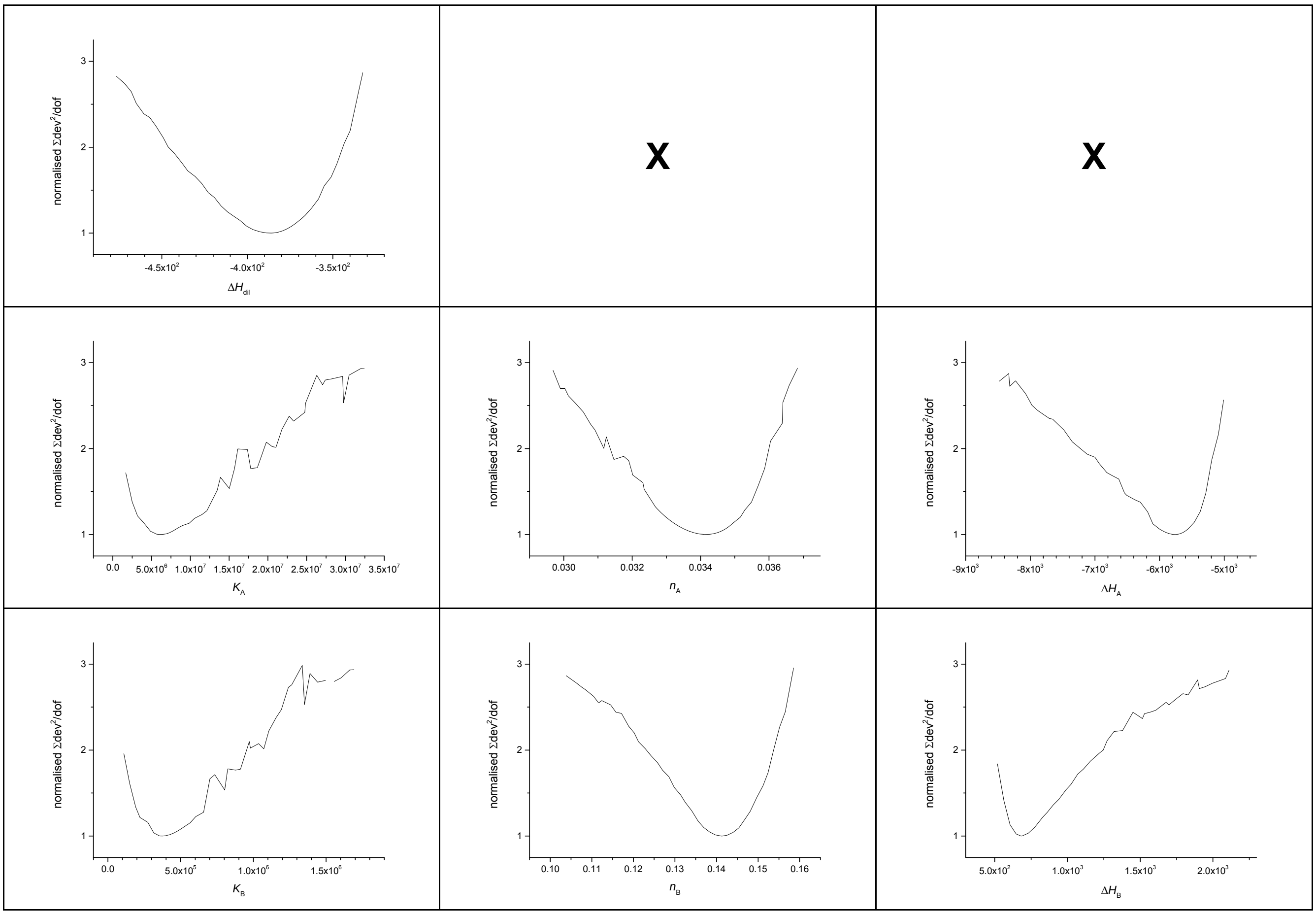

B: $(dGdC)_{12} \cdot (dGdC)_{12}$

Fitted data

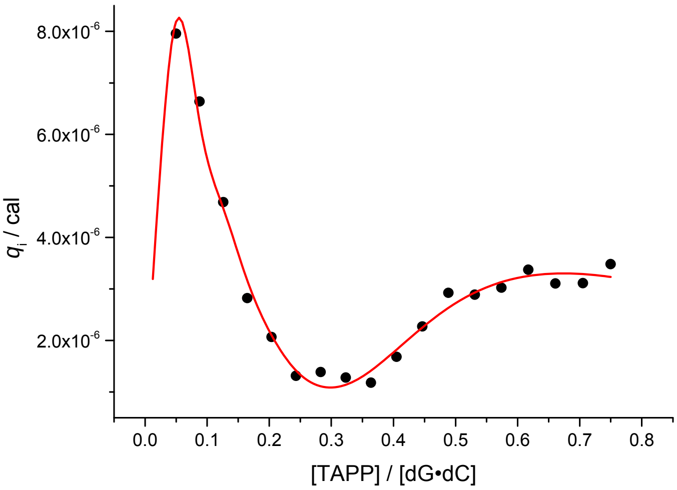

Normalised $\Sigma dev^2/dof$ as a function of optimisable parameter values; parameter values for which the normalised $\Sigma dev^2/dof$ is less than 2 should be considered within error margins.

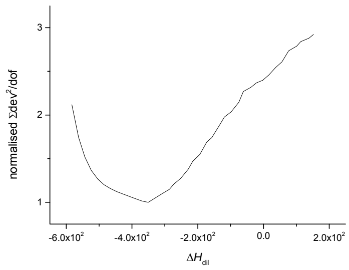	**X**	**x**
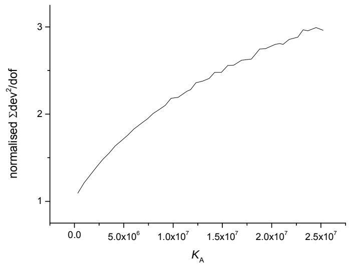	**0.0833**	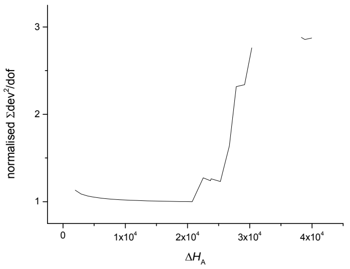

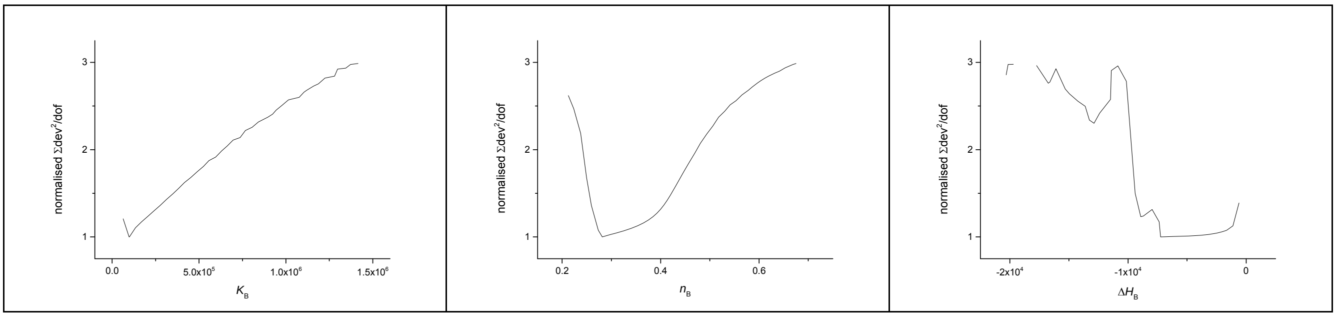

ΔH_A and ΔH_B are ill defined as a result of covariance between these two parameters as illustrated by the contour plot of the normalised $\Sigma dev^2/dof$ as a function of the values of ΔH_A and ΔH_B:

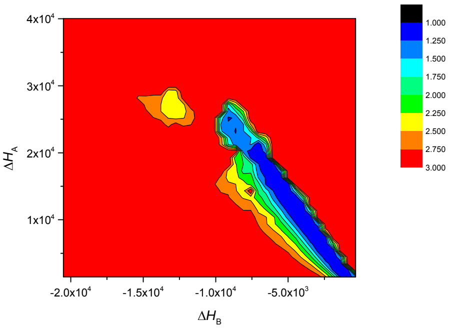

C: 22AG

Fitted data

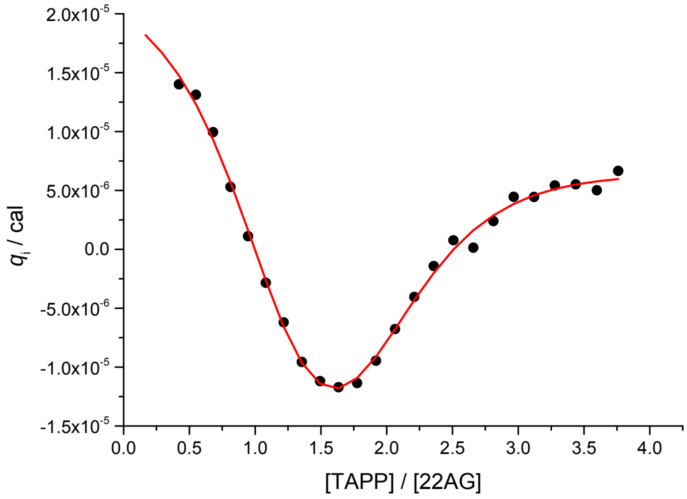

Normalised Σdev²/dof as a function of optimisable parameter values; parameter values for which the normalised Σdev²/dof is less than 2 should be considered within error margins.

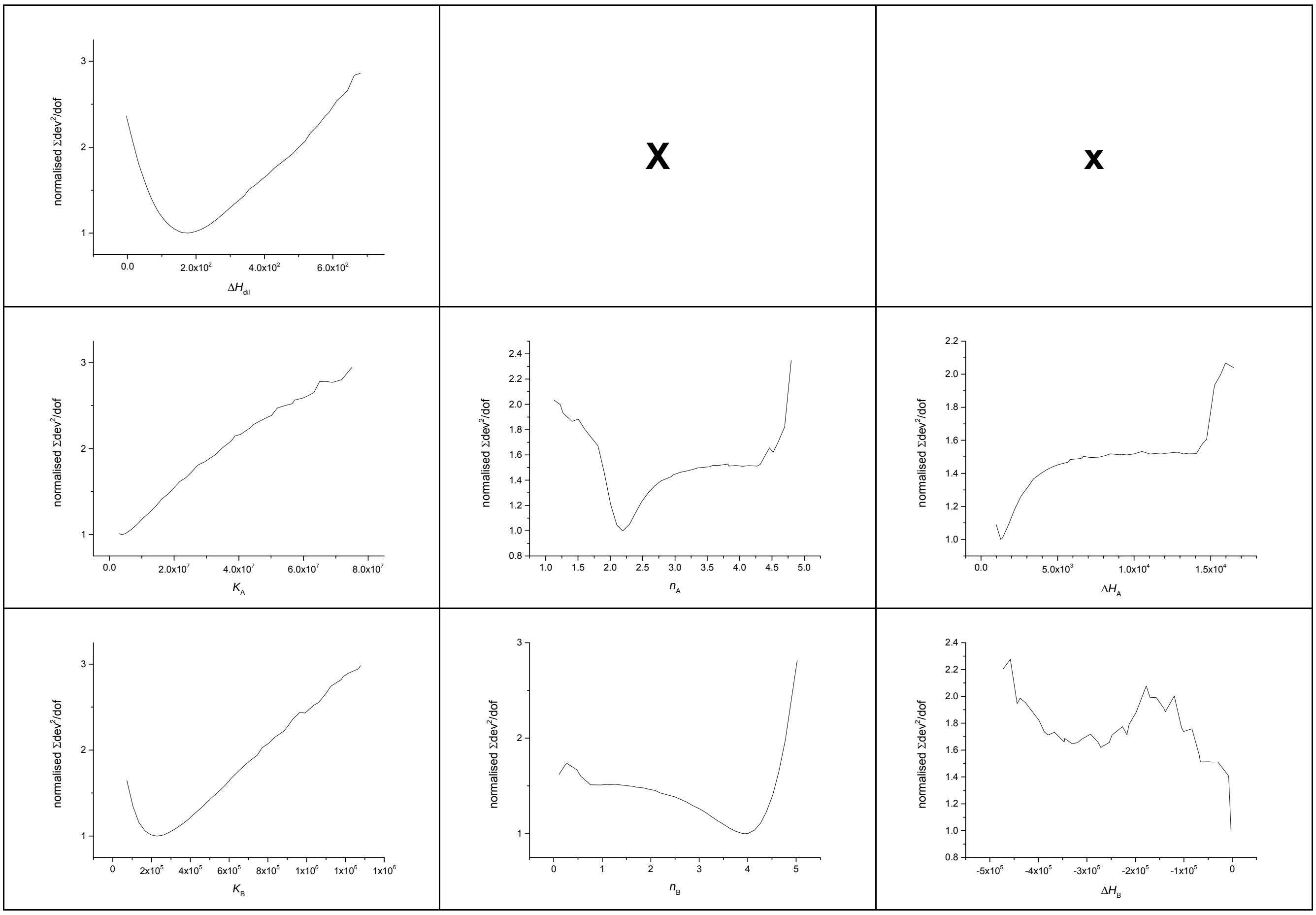

D: c-myc

Fitted data

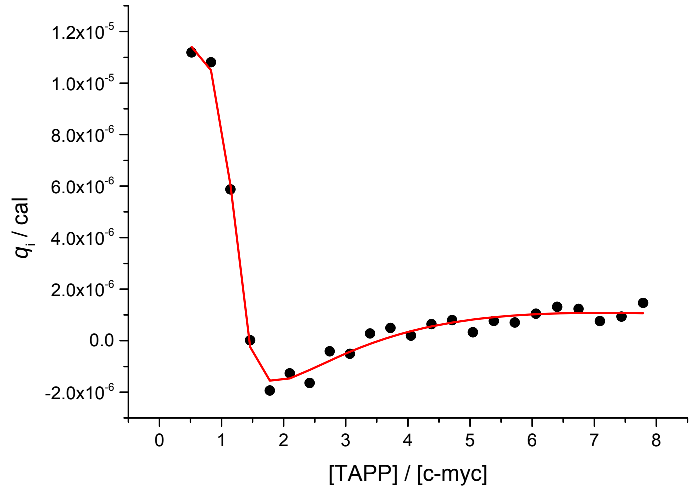

Normalised $\Sigma dev^2/dof$ as a function of optimisable parameter values; parameter values for which the normalised $\Sigma dev^2/dof$ is less than 2 should be considered within error margins.

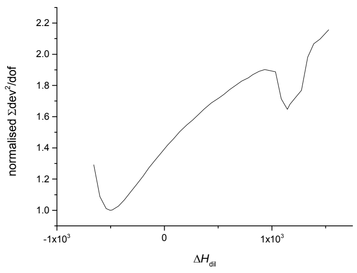	**X**	**x**
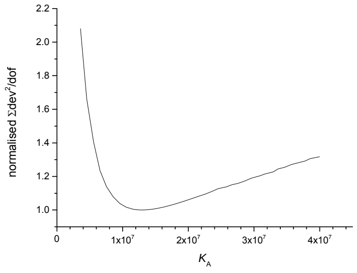	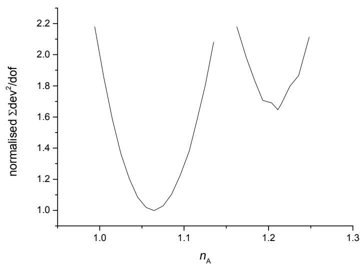	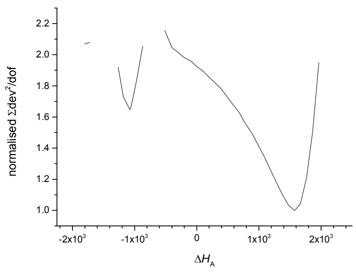
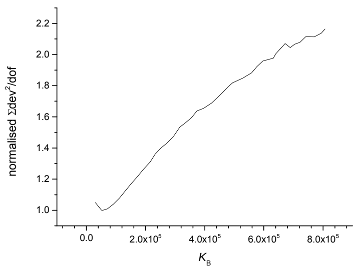	**2**	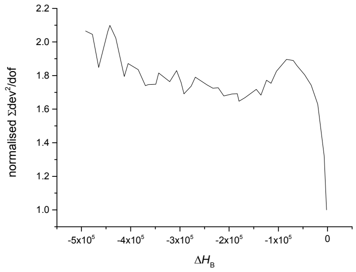

S1 Docking studies

Structures were obtained from the nucleic acids database [2-3] using the sequence definitions as in Table S1.

Table S1: Sequence definitions used to search nucleic acid structures		
22AG		(DA)(DG)(DG)(DG)(DT)(DT)(DA)(DG)(DG)(DG)(DT)(DT)(DA)(DG)(DG)(DG)(DT)(DT)(DA)(DG)(DG)(DG)
c-myc		(DT)(DG)(DA)(DG)(DG)(DG)(DT)(DG)(DG)(DG)(DT)(DA)(DG)(DG)(DG)(DT)(DG)(DG)(DG)(DT)(DA)(DA)

Selection of 22AG structures

Using the sequence definition above for 22AG, 16 structures were found, of which 6 structures contained more bases than the minimum sequence. These structures were ignored. Of the remaining 10 structures, 2 were nucleic acid structures without bound ligands (PDB 1KF1 and 143D) and 8 were nucleic acid structures with bound ligands (PDB 2MCO, 2MCC, 3UYH, 4FXM, 4G0F, 3R6R, 3SC8 and 3T5E). To include, to an extent, the effect of the flexibility of the 22AG structure, docking studies were carried out using all 10 reported relevant structures as targets. In addition to the reported parallel and antiparallel structures for unmodified 22AG, we also used the mixed-hybrid structure 2E4I for a modified 22AG sequence as a target in our docking studies to reflect the fact that 22AG is expected to adopt this mixed-hybrid conformation under our experimental conditions.

Selection of c-myc structures

For c-myc, 2 structures were found (PDB 2L7V and 1XAV) and both were used as targets.

Pre-docking treatment of quadruplex structures

Where the structure includes multiple models, only the first model was used as a target for docking. Where the structure contained a ligand and/or water molecules, these were removed using UCSF Chimera. Ligands and/or water molecules were removed by selecting the ligand and/or water molecules, inverting the selection, and saving the resulting selection as a PDB file. Where a structure contained 3 potassium ions, with one potassium ion on the outside of the stack of three tetrads, this cation was removed.

TAPP 1 PDBQT file

The generation of the PDBQT file for TAPP **1** using AutoDockTools resulted in a predominantly rigid structure, with only the bonds indicated in red in Scheme S1 being rotatable.

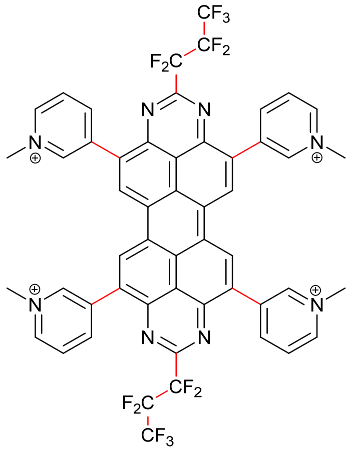

Scheme S1

S2 Comparison of interaction parameters from spectroscopic and calorimetric studies

duplex-[$(dGdC)_{12}$•$(dGdC)_{12}$]

Following data fitting, the simulated annealing trajectories were analysed to obtain plots of the normalised sum over square deviations divided by degrees of freedom ($\Sigma dev^2/dof$) as a function of the equilibrium constants K_A and K_B and as a function of the equilibrium constant K_A and the interaction enthalpy ΔH_A (Figure S5).

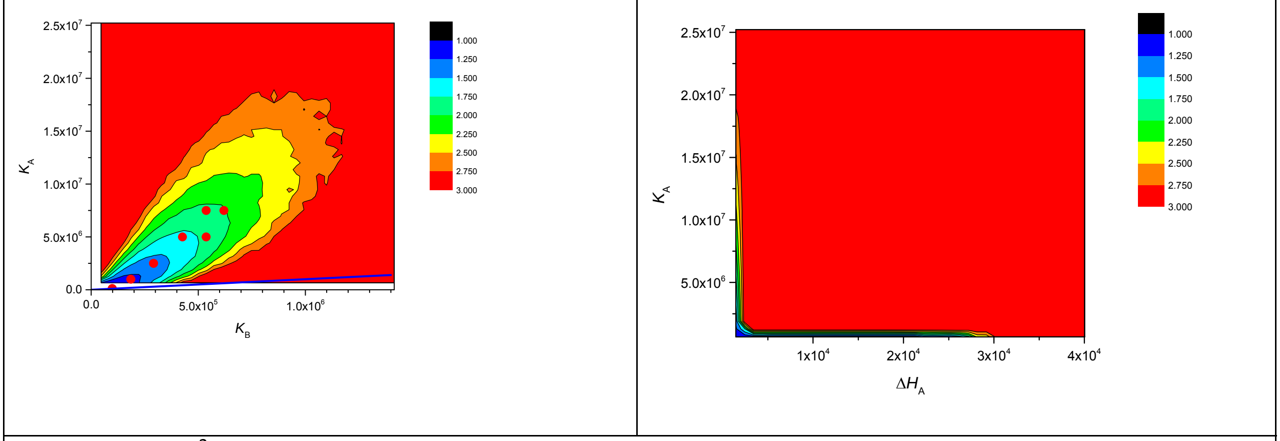

Figure S5: $\Sigma dev^2/dof$) as a function of the equilibrium constants K_A and K_B (*Left*) and as a function of the equilibrium constant K_A and the interaction enthalpy ΔH_A (*Right*). Red dots indicate combinations of K_A and K_B for which free ligand concentrations in the spectroscopic titrations have been simulated. The black line represents $K_A = K_B$.

Figure S5 shows that K_A and K_B are correlated in the fit to the calorimetric data and that the combination of optimised values ($K_A = 1.2\times10^5$ and $K_B = 0.98\times10^5$) corresponds to a lower range of the possible values for K_A and K_B. The optimised value for K_A is in fact low in combination with a very large optimised value for ΔH_A. This is a not infrequent outcome for data showing a $\Sigma dev^2/dof$ as a function of K_A and ΔH_A as in Figure S5 (Right) where very low values of K_A tend to correlate with compensating high values of ΔH_A.

We explored the link between the equilibria identified from the calorimetric data and the single apparent equilibrium quantified using the spectroscopic data. To this effect, we simulated the concentrations of the various species present in solution during the spectroscopic titrations according to combinations of values of K_a, n_A, K_B and n_B identified as reasonable in the analysis of the calorimetric data. These combinations have been identified by the red dots in Figure S5. We then fitted the multiple independent binding sites model to the concentration of the free ligand because we analysed a disappearing spectroscopic signal in our data analysis and the decreases of this signal is dominated by the removal of the free ligand from solution during the titration. The results of this procedure are summarised in Table S2.

Table S2: Apparent equilibrium constants representing fits of the multiple independent binding sites model involving one type of binding sites to data simulated for concentrations as used in the spectroscopic titrations, using combinations of values of K_a, n_A, K_B and n_B identified as reasonable in the analysis of the calorimetric data.

two types of binding sites model (from calorimetry)				apparent single type of binding sites model	
K_A / 10^6 M^{-1}	n_A (binding sites / basepair)	K_B / 10^6 M^{-1}	n_B (binding sites / basepair)	K_{app} / 10^6 M^{-1}	n_{app} (binding site size)
0.12	0.08333	0.098	0.281	0.105	2.81
1.0	0.08333	0.185	0.374	0.377	2.82
2.5	0.08333	0.291	0.393	0.639	2.76
5.0	0.08333	0.426	0.401	0.891	2.62
5.0	*0.08333*	*0.537*	*0.405*	*0.983*	*2.44*
7.5	0.08333	0.537	0.405	1.063	2.53
7.5	0.0833	0.62	0.397	1.150	2.48

Table S2 shows that the apparent stoichiometry of the interaction that would be obtained from the simulated spectroscopic data is typically between 2.5 and 3.0, in good agreement with the observations from the spectroscopic titrations. In addition, it is clear that for higher values of K_A the apparent affinity constant increases to the same order of magnitude as the value observed from the spectroscopic titrations. If we also take the spectroscopic data into account, it is therefore very likely that the values for the affinity constants of **1** for GC are rather higher than the values obtained from the analysis of the calorimetric data alone. We favour values for K_A around 5×10^6 M^{-1} and values for K_B of around 5×10^5 M^{-1}. These values are further associated with a ΔH_A of +0.7 kcal mol^{-1} and a ΔH_B of -0.5 kcal mol^{-1}. Both these values fit well with the enthalpy changes observed for the interactions of **1** with the other nucleic acid structures.

22AG

As for duplex-[$(dGdC)_{12}$•$(dGdC)_{12}$], combinations of reasonable values of K_A and K_B were identified (Figure S6).

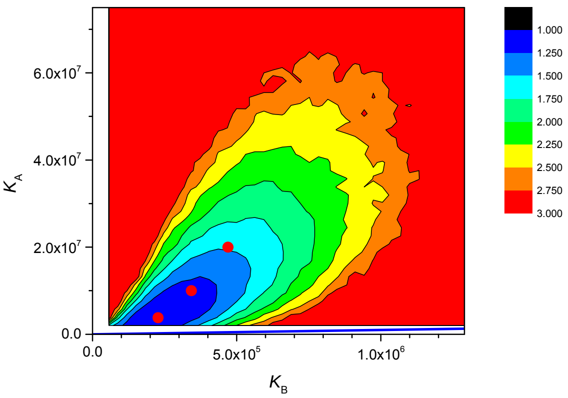

Figure S6: $\Sigma dev^2/dof$) as a function of the equilibrium constants K_A and K_B. Red dots indicate combinations of K_A and K_B for which free ligand concentrations in the spectroscopic titrations have been simulated. The black line represents $K_A = K_B$.

For three reasonable combinations of K_A and K_B (red dots in Figure S6), we simulated the concentrations of the various species present in solution during the spectroscopic titrations. We then fitted the multiple independent binding sites model to the concentration of the free ligand because we analysed a disappearing spectroscopic signal in our data analysis and the decreases of this signal is dominated by the removal of the free ligand from solution during the titration. The results of this procedure are summarised in Table S3.

Table S3: Apparent equilibrium constants representing fits of the multiple independent binding sites model involving one type of binding sites to data simulated for concentrations as used in the spectroscopic titrations, using combinations of values of K_a, n_A, K_B and n_B identified as reasonable in the analysis of the calorimetric data.

two types of binding sites model (from calorimetry)				apparent single type of binding sites model	
K_A / 10^6 M^{-1}	n_A (ligand / quadruplex)	K_B / 10^6 M^{-1}	n_B (ligand / quadruplex)	K_{app} / 10^6 M^{-1}	n_{app} [T]/[TAPP]
3.80	2.19	0.228	3.95	1.946	1.22

10.0		2.13		0.343		4.22		3.477		1.26
20.0		2.11		0.470		4.34		4.700		1.32

Figure S5a: CD-spectrum of 22AG (2 µM) in buffer (25 mM 3-(*N*-morpholino)-propanesulfonic acid (MOPS), 100 mM KCl, 1 mM EDTA, pH 7.1) at 20 °C.

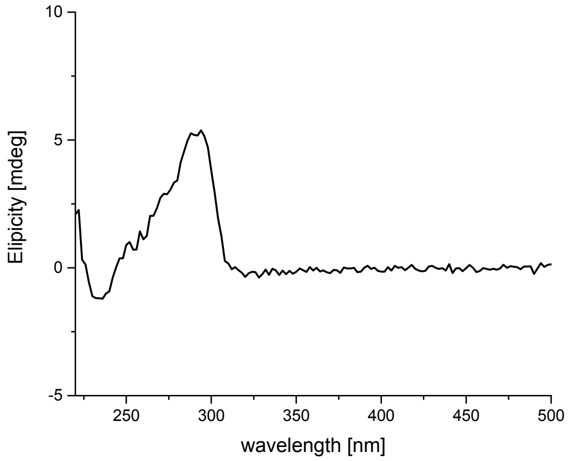

Figure S5b: CD-spectra for the titration of 1 (4 x 10^{-6} M) with 22AG (0 - 4 eq.) in buffer (25 mM 3-(*N*-morpholino)-propanesulfonic acid (MOPS), 100 mM KCl, 1 mM EDTA, pH 7.1)

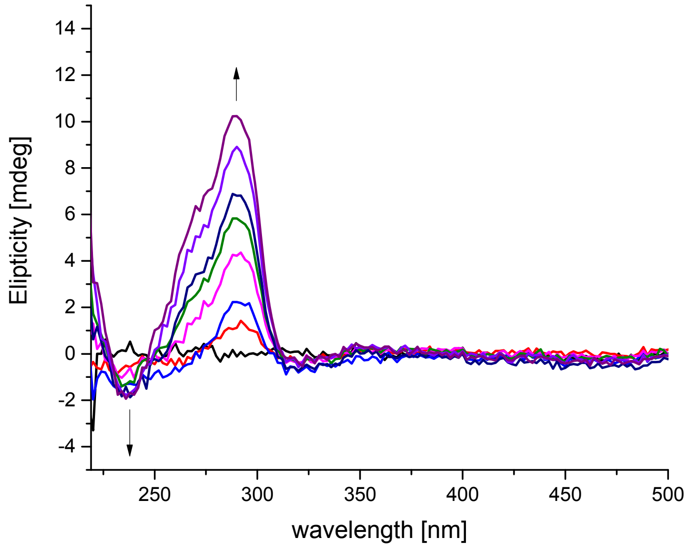

S3 References

[1] N. J. Buurma, I. Haq, *J. Mol. Biol.* **2008**, *381*, 607–621.
[2] H. M. Berman, W. K. Olson, D. L. Beveridge, J. Westbrook, A. Gelbin, T. Demeny, S. H. Hsieh, A. R. Srinivasan, B. Schneider, *Biophys. J.* **1992**, *63*, 751–759.

[3] B. Coimbatore Narayanan, J. Westbrook, S. Ghosh, A. I. Petrov, B. Sweeney, C. L. Zirbel, N. B. Leontis, H. M. Berman, *Nucleic Acids Res.* **2014**, *42*, D114–D122.
